# Genomic Offsets Predict Survival With Low Accuracy in a Marine Common Garden

**DOI:** 10.1111/mec.70457

**Published:** 2026-07-04

**Authors:** Camille A. Rumberger, Madeline G. Eppley, Kiran Bajaj, Nicole Mongillo, Shelley Katsuki, Jessica A. Small, Katie E. Lotterhos

**Affiliations:** ^1^ Marine Science Center Northeastern University Nahant Massachusetts USA; ^2^ Gloucester Marine Genomics Institute Gloucester Massachusetts USA; ^3^ Virginia Institute of Marine Science Gloucester Point Virginia USA

**Keywords:** adaptation, climate change, *Crassostrea virginica*, genetic–environment interactions, genomic offset, predictive ecological genomics

## Abstract

Predicting how populations will respond to climate change is an urgent priority in evolutionary and conservation biology. Genetic and environmental influences have long been known to impact responses to shifting climates, giving rise to the development of genomic offset methods that attempt to predict the degree of maladaptation following environmental change. Although these methods have the potential to improve our understanding of populations' responses to climate change, questions remain about how well they predict fitness in a diversity of systems. Here, we pair genomic offsets with a large‐scale common garden experiment to investigate how fitness of a marine mollusc, *Crassostrea virginica* , varies across environmental gradients and to validate offset predictions against empirical fitness. We find that at two common garden sites in the Chesapeake Bay, Northern oysters had the lowest survival and growth, whereas Southern oysters had similar survival and growth to local wild oysters and selection lines. We compared survival and length to offset predictions, finding higher method performance at the lower salinity, moderate disease site than at the higher salinity, higher disease site. The inclusion of disease in the offset model training additionally impacted offset method performance, though the effect varied between common garden sites, fitness proxies and across methods. Despite a nearly 50% difference in survival across sites, the magnitude of offset predictions to both common gardens was similar, highlighting issues with extrapolating offset predictions across species ranges. Performance of genomic offset methods in this system was not adequate to make real‐world predictions to environmental stressors.

## 1 Introduction

As anthropogenically‐induced climate change accelerates, natural populations are often under pressure to adapt to their changing landscapes. In response to these changes, species can migrate to track their climate niche, respond plastically to tolerate negative climate impacts, evolve to adapt to their new environment, or go extinct (Waldvogel et al. 2020). Given the growing body of literature predicting widespread range contractions and extreme shifts in climate, adaptive evolution will be crucial for species to avoid extinction (Dawson et al. 2011; Smith et al. 2014; Urban 2015). Understanding and predicting adaptation is thus critical to building our understanding of population resilience in changing environmental landscapes. The emerging field of genomic forecasting incorporates bioclimatic modelling approaches with genomics to make such evolutionarily informed predictions (Waldvogel et al. 2020; Lotterhos 2024a). Genomic forecasting, and particularly genomic offset modelling, holds promise for improving predictions of species responses to environmental change, with potential applications in restoration, conservation and management (Rellstab et al. 2021).

Genomic offsets are a subset of genomic forecasting methods that use patterns of genome‐wide allele frequency variation along environmental gradients to estimate the disruption of genotype‐environment associations, and thus local adaptation, following an instant environmental shift (Fitzpatrick and Keller 2015; Rellstab et al. 2016, 2021). These methods are generally thought to be an improvement over species distribution models because they take into account genetic variation across environmental gradients, and thus local adaptation to climate, potentially improving predictions of population responses to climate change (Fitzpatrick and Keller 2015; Capblancq and Forester 2021; Fitzpatrick et al. 2025). Genomic offset methods estimate potential maladaptation of a given population when moved from one environment to another and are used to infer how population fitness will change with that environmental change (Fitzpatrick and Keller 2015; Rellstab et al. 2021; Gain et al. 2023), although this interpretation of their outputs has been debated (Lotterhos 2024a; Ahrens et al. 2025). For these reasons, the use of genomic offsets remains controversial when the link between fitness and predicted offset is not well established (Capblancq et al. 2020; Láruson et al. 2022; Rellstab et al. 2021; Lind et al. 2024).

Previous experiments evaluating genomic offset method performance generally use empirical or simulated common garden experiments to collect data on fitness proxies of experimental individuals or populations (Rhoné et al. 2020; Fitzpatrick et al. 2021; Lachmuth et al. 2023; Lind et al. 2024; Lind and Lotterhos 2025). These ‘ground‐truth data’ are then compared to genomic offset model predictions for the individuals or populations used in the common garden. In order to meaningfully extrapolate offsets across species ranges, they need to be accurate across common gardens in different environments. Genomic offset methods have variable, context‐dependent predictive performance, but studies using genomic offsets to make predictions are far rarer than those assessing their performance (Lotterhos 2024b; Fitzpatrick et al. 2025). Furthermore, these methods have only been evaluated against ground‐truth experimental data in terrestrial systems (Gain et al. 2023; Layton et al. 2024). No validation studies exist for marine systems, which have fewer barriers to gene flow and less steep selective gradients (Sanford and Kelly 2011), despite the increasing use of genomic offsets in marine species to make predictions (reviewed in Layton et al. 2024). Many marine species have also faced widespread disease outbreaks in recent years, exacerbated by rapid climate change (Burge et al. 2014; Randall and van Woesik 2015; Rowley et al. 2024). Despite this, previous applications and validations of genomic offsets have not yet considered spatially heterogeneous biotic drivers of selection such as disease. Validation experiments in marine seascapes, particularly those faced with strong biotic pressures, are needed to better understand the limitations of genomic offsets for predicting species‐ and population‐level responses to climate change.

The eastern oyster, *Crassostrea virginica* , is a reef‐building mollusc that has been shown to adapt to gradients in salinity, temperature and disease pressure (Frank‐Lawale et al. 2014; Proestou et al. 2016; Griffiths et al. 2021). This species has a long history of economic, cultural and ecological importance, particularly in the Chesapeake Bay, where it supports a large fishery and provides a wealth of ecosystem services (Wells 1961; Grabowski et al. 2012; Zu Ermgassen et al. 2012). As sessile organisms, oysters in the Chesapeake are subject to myriad biotic and abiotic environmental gradients that impose strong selective pressures. Temperatures in the Bay range from 1°C to 30°C (Ding and Elmore 2015), salinity from 0 to 35 ppt (Pritchard 1952), and there is significant seasonal disease pressure from Dermo (caused by *Perkinsus marinus*) and MSX (caused by *Haplosporidium nelsoni* ) (Andrews 1979; Carnegie and Burreson 2011; Kachmar et al. 2025).

To understand the utility of genomic offsets in predicting real‐world fitness consequences, we used large‐scale common garden experiments to ground‐truth genomic offset predictions. Specifically, we aim to address:

*Q1*: How do fitness metrics of oysters from different source environments vary from a moderate salinity, high disease site to a low salinity, moderate disease site?

*Q2*: How well do different genomic offset methods predict observed patterns of fitness metrics in each common garden?

*Q3*: What effect does the biotic disease landscape have on genomic offset prediction and model performance?

We raised oysters sourced from across their native range in a 2‐year common garden experiment where we collected fitness proxy data of eastern oyster populations at both a moderate salinity, high disease site and a low salinity, moderate disease site. We leverage a seascape genomic dataset consisting of 630 individuals from 32 sites to train genomic offset models, incorporating both biotic and abiotic selective pressures into the training. We then use these trained models to predict offset of our six experimental wild populations and two selection lines to our two common garden sites and compare ground‐truth fitness proxies in the common garden to the offset predicted by the genomic offset models. This study represents the first test of genomic offsets in a marine species and is additionally the first to consider adaptation to biotic pressures.

## 2 Methods

### 2.1 Common Garden Experiment

#### 2.1.1 Experimental Design

**FIGURE 1:**
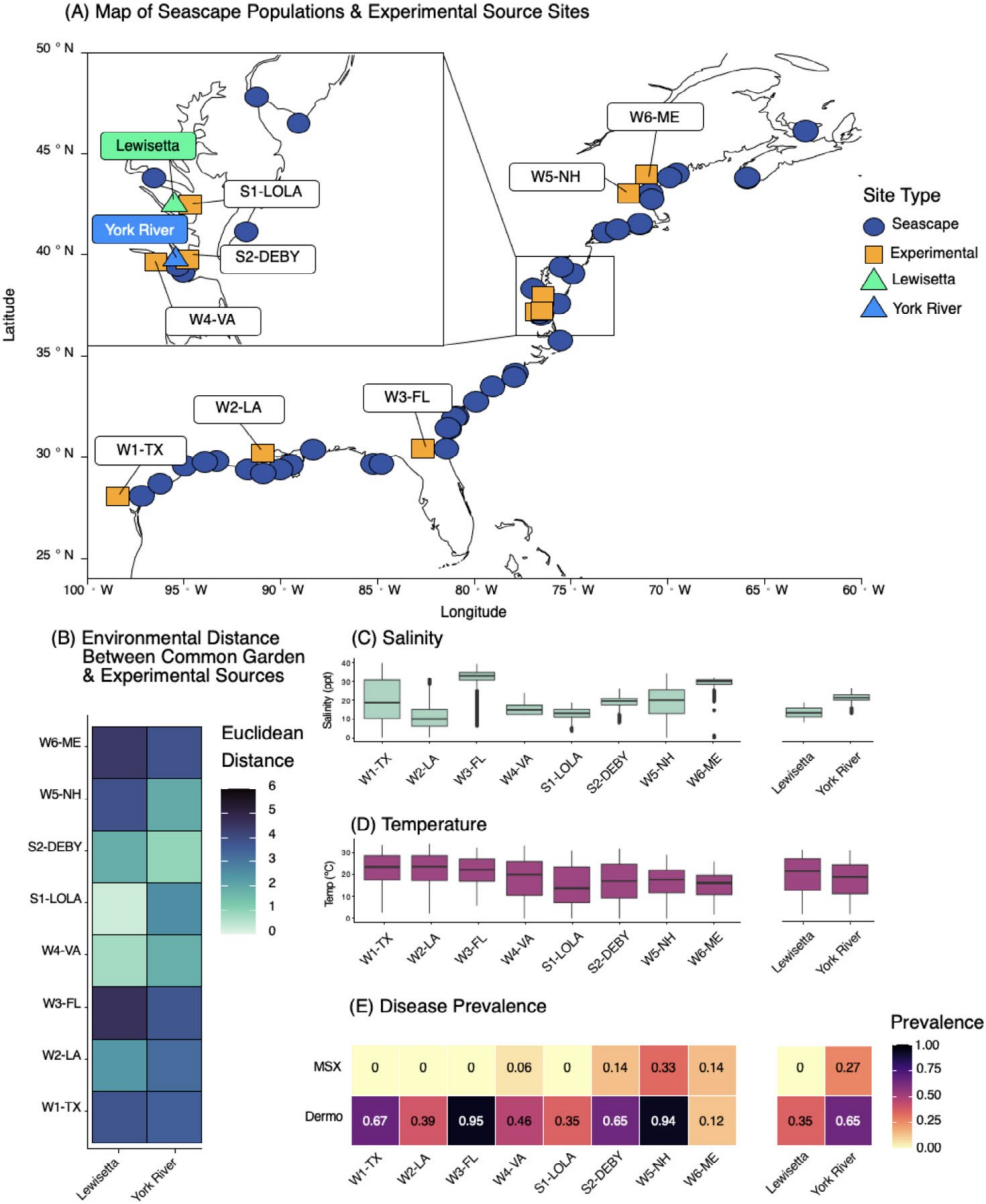
Seascape, experimental, and common garden sites. (A) Map of seascape sites used for model training (blue circles) and source sites for populations used in experiment and for model predictions (orange squares), with inset of the Chesapeake region, with common garden sites Lewisetta (mint triangle) and York River (blue triangle). (B) Environmental distance between experimental source populations and common garden sites. (C) Salinity (ppt), (D) temperature (°C) and (E) median disease pressure (prevalence) for experimental source populations (left) and common garden sites (right).

Adult oysters from six wild populations and two selection lines were spawned to create juvenile oysters, which were then deployed for 2 years as two common gardens in the Chesapeake Bay: one location was in the Coan River, VA, herein ‘Lewisetta’, and the other in the York River, VA. Lewisetta is characterized by relatively low salinity (average 8–15 ppt) and moderate prevalence of the oyster parasites Dermo and MSX (Figure 1C,E, Figures S1 and S2; Frank‐Lawale et al. 2014). The York River, in contrast, has moderate salinity (average 15–23 ppt) and higher disease pressure (Figure 1C,E, Figures S1 and S2). At both sites, mean daily temperatures range from 5°C to 30°C, with freezing common in the intertidal zone in winter months (Figure 1D, Figure S2).

#### 2.1.2 Experimental Population Sourcing

Between September and November of 2022, 50–80 oysters were collected from six sites distributed along the eastern oyster's native range (see Table 1) then shipped live to the Virginia Institute of Marine Science's Aquaculture Genetics & Breeding Technology Center (ABC) where the animals were held in a quarantine system until late winter 2023, and subsequently conditioned in a closed‐loop system for approximately 8 weeks prior to spawning. Two additional proprietary ABC brood stock lines, LOLA and DEBY, were also used in this experiment. These lines have been selected for growth and survival at the common garden sites, and so we included them as populations that would in theory be adapted to local conditions. DEBY was established by consolidating several Delaware Bay lines (77%) with Mobjack Bay (14%) and Louisiana oysters (9%) over 10 years, and has been under selection for York River conditions for eight generations. LOLA was established later by introgressing Louisiana oysters (80%) with DEBY oysters (20%), and has been under selection to Lewisetta conditions for at least four generations (Ragone Calvo et al. 2003; Frank‐Lawale et al. 2014; Puritz et al. 2022). In accordance with their selection history, DEBY has historically performed better in the moderate salinity in the York River and LOLA in the low salinity conditions in the Lewisetta (Roy and Kirchner 2000; Proestou et al. 2016).

**TABLE 1:** Populations included in the common garden experiment.

Relative geographic region	Ancestral group	Location	Site abbreviation	Latitude	Longitude
South	Gulf	Sister Lake, LA	W1‐LA	29.239925	−90.911362
South	Gulf	Copano Bay, TX	W2‐TX	28.096	−97.174
South	Atlantic	Timucuan Preserve, FL	W3‐FL	30.44003	−81.43638
Local	Atlantic	Deep Water Shoal (James River), VA	W4‐VA	37.14888	−76.63554
North	Atlantic	Great Bay, NH	W5‐NH	43.053746	−70.911544
North	Atlantic	Hog Island, ME	W6‐ME	44.0133	−69.541689
Local	Selected	LOLA (grown at Lewisetta)	S1‐LOLA	37.98030	−76.46190
Local	Selected	DEBY (grown at York River)	S2‐DEBY	37.24728	−76.49937

*Note:* Table of oyster populations included in the common garden experiment.

#### 2.1.3 Conditioning, Spawning and Larval Rearing

Oysters were held at ABC in a flow‐through conditioning system. During conditioning, algal feed levels and temperature were manipulated to induce gamete production. In May 2023, parental oysters were measured prior to spawning. For each population, 10 males and 10 females were strip spawned according to standard hatchery protocol and crossed to make larval cultures. Larvae were reared in duplicated 60 L & 200 L tanks containing aerated 1 μm filtered seawater and maintained at 25°C–27°C. Larvae were fed a standard ration of live microalgae. At Days 2, 7 and 14, the density of larval cultures was reduced to prevent overcrowding. Eyed larvae were set on microcultch in a downwelling system and reared until 1 mm in shell length, then transitioned to an upwelling nursery system until 5–10 mm in shell length.

#### 2.1.4 Field Deployment & Monitoring

Experimental seed was deployed at two field sites, Lewisetta (37.98030, −76.46190) and York River (37.24728, −76.49937), in July 2023. For each experimental source population, approximately 1200 oysters per group were placed into each of six 4 mm mesh oyster bags, three per common garden. Deployed bags were monitored every 6 months for bag‐level survival and individual‐level length (longest distance from shell hinge to shell edge). These data acted as proxies to track fitness through time. At each monitoring event, bags were removed from water for a standardized period of time and checked for survival. At the first monitoring event, the length and width of 40 oysters per bag were taken, then oysters were tagged with shellfish tags (Hallprint) held in place by coral epoxy for future identification. These tagged oysters were re‐identified and re‐measured at each subsequent monitoring event to track length through time. To reduce density‐dependent growth, bags were thinned to a density of 225 oysters per bag in May 2024. To account for this thinning, survival at each monitoring timepoint (*S*_*t*_) was calculated recursively as the number of live oysters at that monitoring event (alive_*t*_) divided by the number of alive and dead oysters at that time point ((alive + dead)_*t*_), scaled by cumulative survival at the previous monitoring event (*S*_*t* − 1_). Note that for *t* = 1, *S*_*t* − 1_ = 1.

(1) $$S_{t}=\frac{{\text{alive}}_{t}}{{\left(\text{alive}\;+\text{dead}\right)}_{t}}*S_{t-1}$$

### 2.2 Statistical Analysis of Growth and Survival

#### 2.2.1 Analysis of Morphometric Data and Survival of Experimental Oysters

To look for differences in survival and length of oysters from different sources in the common garden (Question 1), we built a set of linear (mixed) models, used ANOVA to determine significance of fixed effects from these models, and examined statistical differences between groups. We confirmed that the following models met the assumptions of normality and homoscedasticity before proceeding. We used Akaike's Information Criterion (AIC) to select the simplest and best‐fitting model. We tested for pairwise differences in the response among groups with post hoc Tukey–Kramer tests.

Specifically, we analysed the response variable of survival (a proportion, one measure per bag) as a function of the explanatory variables of test site, population, and their interactions. We analysed the response variable of length (40 oysters per bag) as a function of the same fixed effects as the survival model while incorporating the random effect of bag using linear mixed models built with lme4 v1.1.37 (Bates et al. 2015). We then used a type III ANOVA to test the significance of each fixed effect in the final model. We estimated degrees of freedom for the length model using the Kenward–Rogers correction implemented in the R package pbkrtest (Kenward and Roger 1997; Halekoh and Højsgaard 2014).

### 2.3 Genomic Offset Modelling

#### 2.3.1 Environmental Data for Modelling

Environmental data were delineated into three datasets: (1) The seascape training dataset consisted of historic environmental data for 32 seascape sites, which was used to train the offset models by establishing the genetic‐environment relationship across the seascape, (2) The experimental source population dataset consisted of historic data for our six wild populations and two selection lines, which was input as the starting environment in the offset model (i.e., the environment being predicted from), (3) The common garden dataset consisted of environmental data for Lewisetta and York River, which were input as the new environment in the offset models (i.e., the environment being predicted to). We developed abiotic and biotic (e.g., disease caused by parasites) environmental datasets for genomic offset modelling, and explored how the inclusion of biotic variables affected the predictive performance of offset models.

For abiotic variables, we included extreme quantiles (10th and 90th quantiles) for temperature (°C) and salinity (ppt). Extreme quantiles were chosen to represent environmental conditions as they presumably capture the extreme conditions likely to impose selective pressures, without suffering from the statistical issues associated with averaging maximums or minimums. Environmental data for the seascape training dataset and experimental source population site‐of‐origin data included the 10th and 90th quantiles for salinity (ppt) and temperature (°C) across as many years as data were available (Eppley et al. 2026; Bajaj et al. 2026). Environmental data for the common garden environment at Lewisetta and York River included the 10th and 90th quantiles for salinity (ppt) and temperature (°C) for only the experimental period.

For biotic (disease) variables, we included population‐level prevalence of the two parasites (disease prevalence). In the seascape training dataset and experimental source population dataset, which were collected during months with historic precedent of peak disease, the prevalence of MSX and Dermo for each population was calculated according to the proportion of individual oysters with each disease as determined by multiplex qPCR assay (see Appendix S1) (Piesz et al. 2022; Ewart and Ford 1993). For the common garden sites, the prevalence of MSX and Dermo in the surrounding environment was determined by using Ray's Fluid Thioglycollate Medium (RFTM) assay on a set of 20 naive ‘sentinel’ oysters deployed on both locations and then assessed monthly during peak disease season (July through November) (Ray 1952; Dungan and Bushek 2015).

All three environmental datasets with abiotic and disease variables were then combined and checked for significant correlation (*r* > 0.75) between all environmental variables. Upon confirmation that no significant correlation existed, data were scaled to have a mean of zero and standard deviation of one using decostand() in the R package vegan v2.7‐2 (Oksanen et al. 2025). The environmental data were then separated into seascape training data, experimental source population site‐of‐origin data, and common garden test site data. We additionally used scaled environmental data to calculate Euclidean environmental distance between each experimental source population's site–of‐origin and common garden sites using vegan's vegdist(), and used environmental distance as an additional measure of offset for comparison.

#### 2.3.2 Genomic Data for Offset Model Training

To train the genomic offset models, we used a seascape genomic dataset of 630 individuals collected from 33 sites distributed across the North American range of *C. virginica* (Eppley et al. 2026). This dataset was thinned for linkage disequilibrium (*r* ^2^ = 0.2) and MAF with the R package bigsnpr v1.12.21 (Privé et al. 2018), retaining a total of 115,185 single nucleotide polymorphisms. We used this entire LD‐thinned data to represent the whole genome, rather than a subset of putatively adaptive loci, as the majority of recent work has shown negligible differences between genomic offset models trained with adaptive loci compared to all loci (Fitzpatrick et al. 2021; Láruson et al. 2022; Capblancq et al. 2023; Lachmuth et al. 2024; Lind et al. 2024; Lind and Lotterhos 2025; but, see Rêgo et al. 2025). Note that the experimental oysters used to estimate fitness proxies for model evaluation were not included in this training dataset.

#### 2.3.3 Population Genetic Statistics

This thinned genomic data were used to characterize pairwise *F*_ST_ among all wild source populations for the common garden experiment, namely W1‐TX, W2‐LA, W3‐FL, W4‐VA, W5‐NH and W6‐ME. Pairwise *F*_ST_ was calculated according to the Weir and Cockerham method (Weir and Cockerham 1984) in the R package hierfstat v0.5.11 (Goudet and Jombart 2024). Other statistics, namely observed heterozygosity (*H*_O_), expected heterozygosity (*H*_S_) and inbreeding coefficient (*F*_IS_) were calculated for each population using the basic.stats() function in hierfstat (Goudet and Jombart 2024).

#### 2.3.4 Genomic Offsets

Genomic offsets were calculated using three methods shown to perform well in experiments and simulation studies (Láruson et al. 2022; Gain et al. 2023; Archambeau et al. 2026; Lind and Lotterhos 2025). These include LEA's genetic gap (LEA_offset_), RDA's genomic offset (RDA_offset_) and gradientForest's genomic offset (GF_offset_) (Gain and François 2021; Capblancq and Forester 2021; Fitzpatrick and Keller 2015). At their core, the genomic offsets output by these methods are a weighted environmental distance, with distance calculated using different approaches to establish the genetic‐environment relationship used to weight each environmental variable for offset prediction.

LEA_offset_ is based on estimates of environmental effect sizes obtained from a latent‐factor‐mixed‐model (LFMM), and the relationship inferred by the LFMM is then used to fit and predict allelic variation at all loci (Gain and François 2021; Gain et al. 2023). LFMM models were built using the R package LEA3 with *K* = 2 latent factors to account for the ancestral split between Gulf and Atlantic coast oyster populations (Frichot and François 2015; Gain and François 2021; Eppley et al. 2026).

RDA_offset_ uses the genetic‐environment relationship inferred from a redundancy analysis (RDA) to calculate an ‘adaptive index’ for both current (sites of origin) and future (common garden sites) environmental data. The difference between these adaptive indices is the genomic offset (Capblancq and Forester 2021). RDA models were trained using vegan's rda(), and constrained axes selected based on visual inspection of eigenvalues (Oksanen et al. 2025). Genomic offset was then calculated in the R package rdadapt (Capblancq and Forester 2021; https://github.com/landscape‐genomics/rdadapt). Offset calculation used *K* = 2 constrained RDA axes, retained based on broken‐stick criterion as established via visual inspection of eigenvalues (Jackson 1993).

GF_offset_ uses regression trees to fit a model of association between individual response variables (loci) to a multivariate set of predictors (climate variables) which can then be used to create a prediction of allelic turnover as a proxy of future population maladaptation, or genomic offset (Fitzpatrick and Keller 2015; Capblancq et al. 2020). The offset model was trained in the R package gradientForest using individual‐level genotype data (Fitzpatrick and Keller 2015).

Genomic offsets were calculated from each experimental source population's site‐of‐origin and each seascape site to each common garden site, though only the former experimental offsets were used for method validation. In order to visualize genomic offset to each common garden across the entire study range, seascape offsets were interpolated using an inverse‐distance weighting algorithm, implemented in the R package gstat v.2.1‐4 (Pebesma 2004; Gräler et al. 2016).

### 2.4 Comparison of Genomic Offset to Experimental Fitness

#### 2.4.1 Comparison of Offset Prediction to Ground‐Truth Fitness Proxies

We then sought to assess how well offset predictions and environmental distance correlated with ground‐truth fitness proxies (Question 2). We quantified performance as the correlation between offset and fitness proxy (bag‐level survival or bag‐mean length) using Kendall's Tau that is robust to violations of the assumption of linearity, and should thus capture any fitness‐offset relationship (Schaeffer and Levitt 1956; Gain et al. 2023; Lotterhos 2024b). If genomic offset or environmental distance from the site of origin to the common garden site is a good proxy for potential maladaptation in the common garden environment, we expect a significant negative correlation between offset and survival or length in the common garden. We calculated these correlations at each common garden in order to determine if the genomic offset methods perform differently in one common garden compared to another, correcting for multiple testing using the Benjamini–Hochberg procedure (Benjamini and Hochberg 1995).

#### 2.4.2 Examining Impact of the Biotic Seascape

To determine the impact that biotic disease variables had on offset prediction and resulting performance of offset models (Question 3), we removed Dermo and MSX prevalence from our environmental dataframe and re‐ran all genomic offset analyses. We then calculated the correlation between these abiotic‐only genomic offset predictions to fitness proxies in the common garden as described above, and evaluated model performance. Finally, we compared both genomic offset predictions and performance made with and without disease data.

## 3 Results

### 3.1 Survival and Length of Oysters Depends on Source Population and Common Garden Site

**FIGURE 2:**
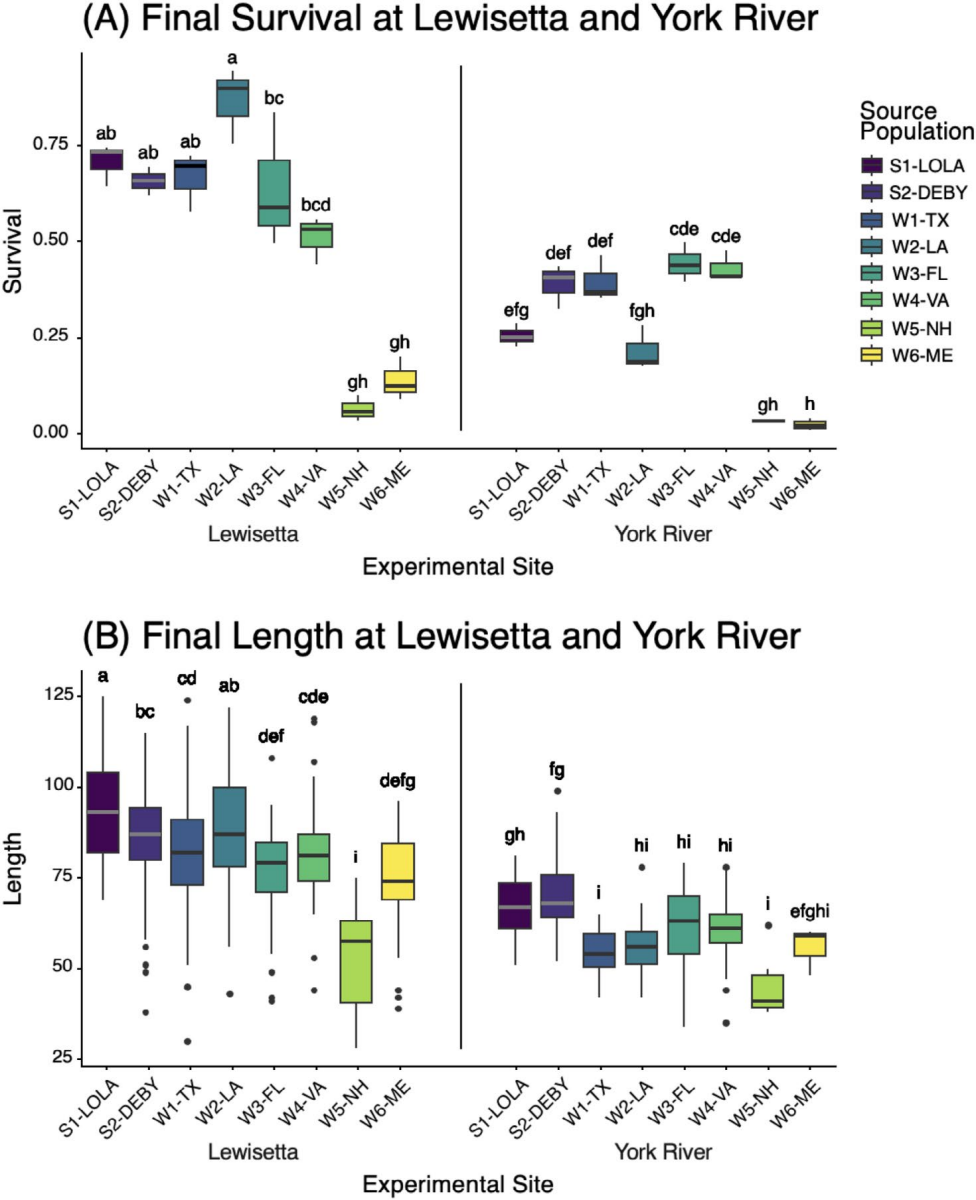
Fitness proxies (A) survival and (B) length at Lewisetta (left) and the York River (right). Boxes are coloured by broodstock group, and letters indicate significant differences between groups.

Linear models revealed that population, common garden site, and their interaction were all significant predictors of survival (Table 2, survival model). Survival was nearly twice as high at the low salinity, moderate disease site Lewisetta (survival_mean_ = 0.520) compared to the moderate salinity, high disease site York River (survival_mean_ = 0.289). Across sites, north Atlantic (W5‐NH, W6‐ME) populations had the lowest survival (File S1). New Hampshire had ~6% survival at Lewisetta and ~2% survival at the York River, whereas Maine had ~14% survival at Lewisetta and ~2% survival at York River. Survival of other populations was more variable across sites. At Lewisetta, the Gulf populations (W1‐LA, W2‐TX) and selection lines (S1‐LOLA, S2‐DEBY) had the highest survival, whereas at the York River, a mix of Gulf populations (W2‐TX, W3‐FL), the local Chesapeake population (W4‐VA) and the selection line (S2‐DEBY) had the highest survival (Figure 2A). As expected, the low‐salinity selection line LOLA had higher survival at Lewisetta than the moderate‐salinity selection line DEBY, whereas DEBY had higher survival than LOLA at the York River, though the difference was not significant at either site.

**TABLE 2:** Type III ANOVA of fitness proxies as a function of population, common garden site, & their interaction.

Source of variation	Sum of squares	df	*F*	Pf(>*F*)
Survival model: Survival ~ Common garden * Population				
Intercept	1.50265	1	291.483	2.460e‐16***
Common garden	0.30673	1	59.500	2.107e‐08***
Population	1.65336	7	45.817	1.126e‐13***
Common garden * Population	0.36995	7	10.252	2.606e‐06***
Residuals	0.14434	28		
Length model: Length ~ Common garden * Population + (1|Bag)				
Intercept		1	2305.003	< 2.2e‐16***
Common garden		1	63.504	1.558e‐09***
Population		7	21.491	1.037e‐08***
Common garden * Population		7	3.942	0.001981**
Residuals		63		

*Note:* Type III ANOVA results for models examining the response variables of (i) survival and (ii) length as a function of common garden test site, population, and their interactions. Survival was analysed using a linear model, whereas length was analysed using a linear mixed model incorporating the random effect of bag ID. Residuals of the length model were assessed using Kenward–Rogers corrected degrees of freedom. Significance is indicated by * *p* < 0.05, ** *p* < 0.01, *** *p* < 0.001.

Length was similarly explained by population, common garden site, and their interaction (Table 2, length model). Oysters were on average larger at Lewisetta (mean_length_ = 83.11 mm) than at the York River (mean_length_ = 62.29 mm) (Figure 2B). The mean length of oysters from different populations was generally not significantly different within common garden sites, with the exception of New Hampshire, which was smaller than other populations across both common gardens (Figure 2B). Pairwise comparison revealed that W2‐LA and S1‐LOLA were largest at Lewisetta, whereas S2‐DEBY was largest at the York River (Figure 2B, File S2).

### 3.2 Characterizing the Genetics of Experimental Populations

Pairwise FST values distinctly group Gulf (W1‐TX, W2‐LA) and Atlantic (W3‐FL, W4‐VA, W5‐NH, W6‐ME) populations, reaching a maximum of 0.156 between W4‐VA and both W1‐TX and W2‐LA (Figure S4). Florida (W3‐FL) had both the highest observed (*H*_O_ = 0.223) and expected (*H*_S_ = 0.269) heterozygosity, which ranged from 0.211 to 0.223 and from 0.252 to 0.269, respectively across all populations (Table S2). Inbreeding (*F*_IS_) was positive for all populations examined (Table S2). The lowest values were observed in the New Hampshire (W5‐NH, *F*_IS_ = 0.135) and Maine (W6‐ME, *F*_IS_ = 0.132) populations, and the highest value was observed in Florida (W3‐FL, *F*_IS_ = 0.158).

### 3.3 Genomic Offset Predictions Vary Across Methods

**FIGURE 3:**
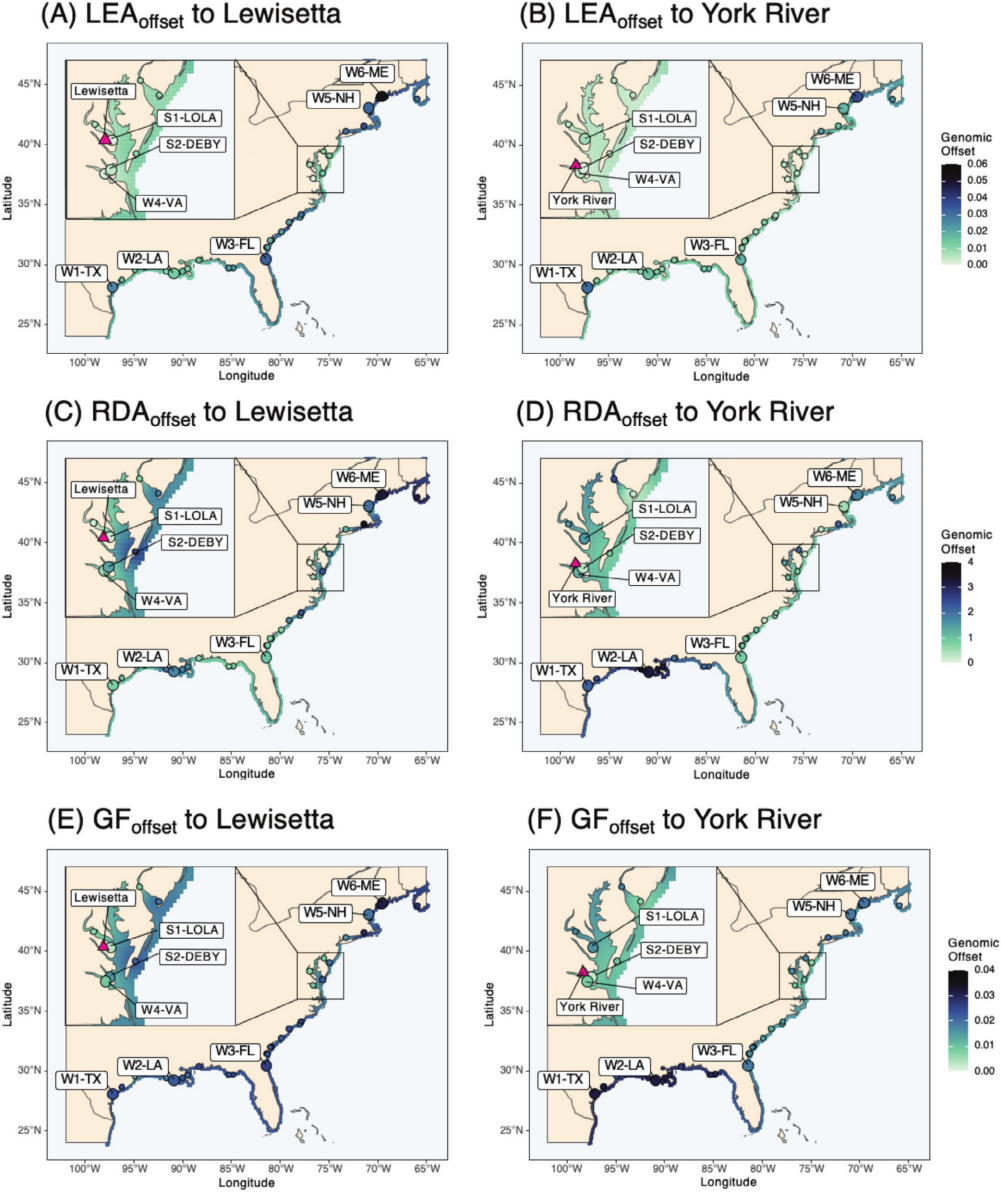
Genomic offsets of oyster populations across the sampled seascape. Each row represents a different genomic offset method ([A, B] LEA_offset_, [C, D] RDA_offset_, [E, F] GF_offset_), whereas each column represents a different common garden site (Lewisetta A, C, E; York River B, D, F). Note that the magnitude of genomic offset differs by method, and plots are coloured according to the range of offset values recovered by each method. Larger labelled points indicate experimental source populations, whereas smaller unlabeled points represent sites used for seascape model training.

As expected, LOLA and DEBY had the lowest offsets to their local sites (Lewisetta and York River, respectively) across all methods. Of the wild populations, W6‐ME consistently had the highest offset to Lewisetta across methods (Figure 3, left column), but no population was consistently predicted to have the highest offset to the York River (Figure 3, right column). The local Virginia population (W4‐VA) was consistently found to have the lowest or second lowest offset of any wild population at both Lewisetta and York River (Figure 2).

Genomic offset of other populations was more variable across methods, particularly at York River (Figure 3, File S4). The three Southern populations from Texas (W1‐TX), Louisiana (W2‐LA) and Florida (W3‐FL) were predicted to have relatively low offset at Lewisetta by LEA, but relatively high offset by gradientForest (Figure 2, left column). At the York River, LEA predicted low offset for these populations, but RDA and gradientForest predicted offsets on par with or even higher than the Maine (W6‐ME) population (Figure 3, right column). Correlations between offsets predicted by different methods and between offset and environmental distance ranged from 0.62 to 0.92 and were not always significant, indicative of potentially problematic differences in interpreting genomic offset predictions made using only one method (Figure S7). Critically, there was also substantial variation in the magnitude of genomic offset predicted by different methods, at times by multiple orders of magnitude. The magnitude of RDA_offset_ was highest, ranging from 0.302 to 3.193, whereas LEA_offset_ had the lowest minimum value, ranging from 3.008e‐04 to 5.240e‐02, and GF_offset_ had the lowest maximum value, ranging from 2.759e‐03 to 3.154e‐02 (Figure 3, Files S3 and S4). This large difference in predictions between methods suggests that the magnitude of genomic offset may be less informative than the relative ranking of populations.

### 3.4 Genomic Offsets Predict Experimental Fitness Proxies With Low Accuracy

**FIGURE 4:**
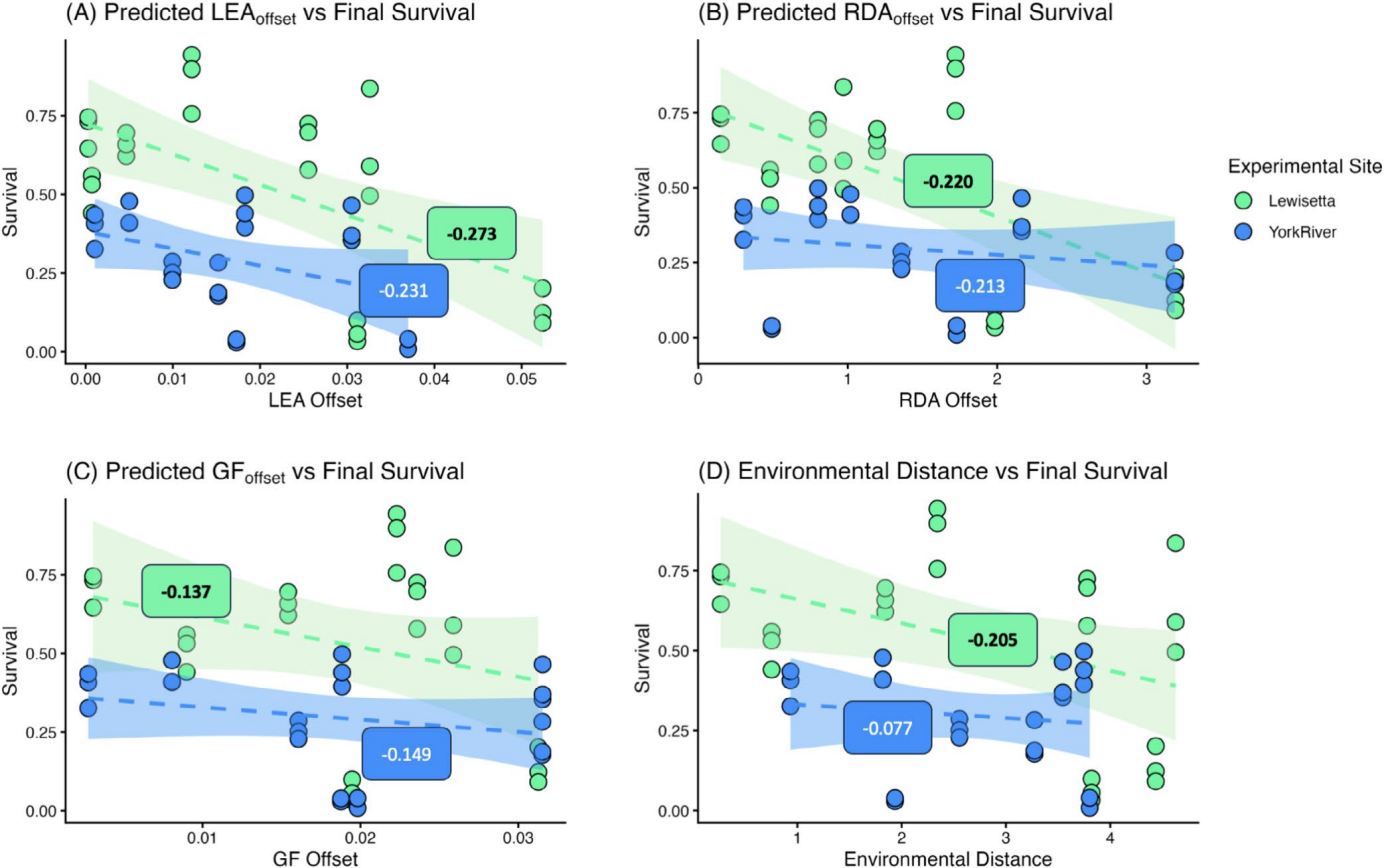
Relationship between survival and genomic offset (A–C) or environmental distance (D) including disease in the model. Each point represents survival in a different bag. Lewisetta is shown in green, whereas York River is shown in blue. Kendall's Tau for each site is shown in boxes. Note that these graphs represent analyses conducted without the selection lines S1‐LOLA and S2‐DEBY.

**FIGURE 5:**
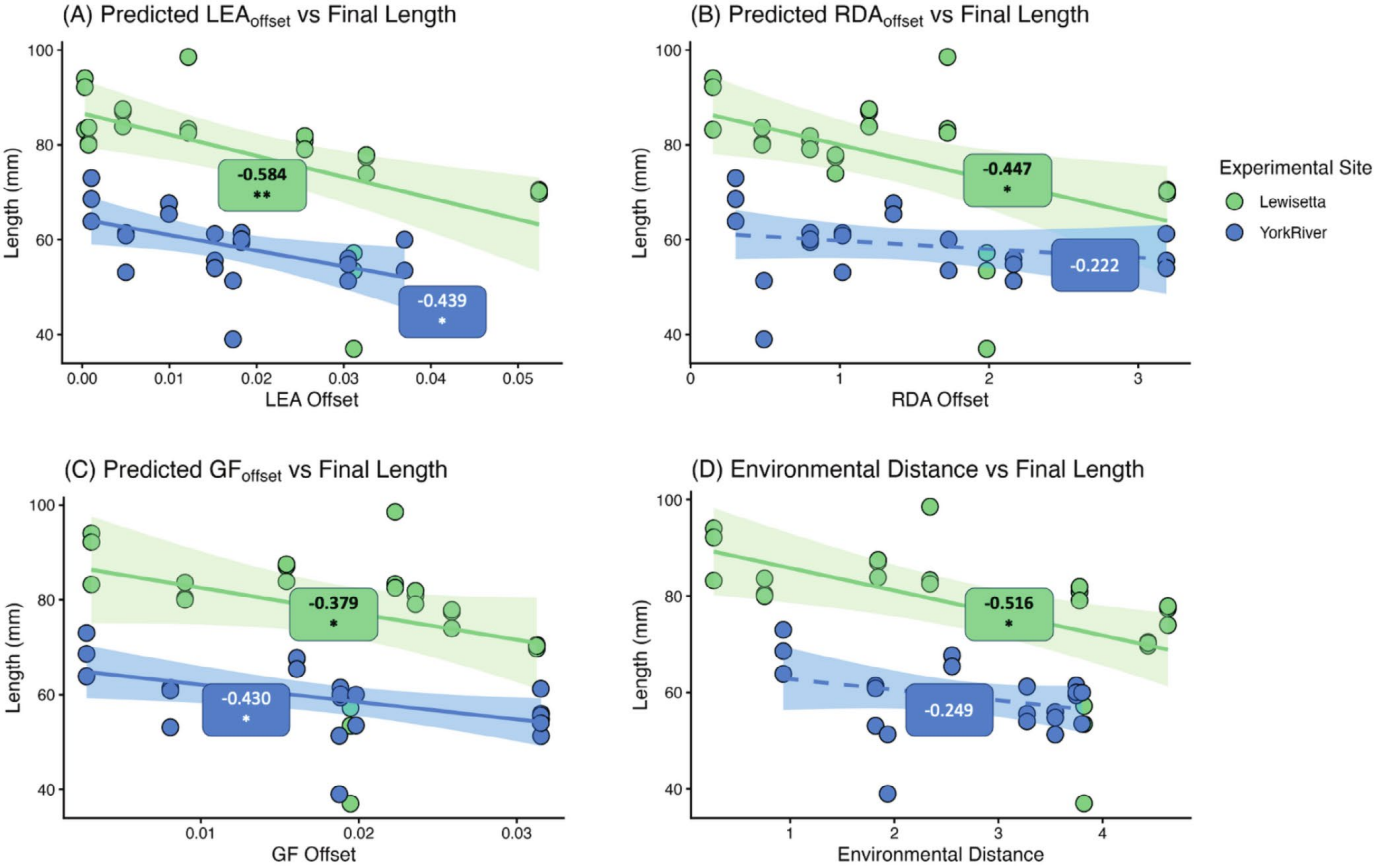
Relationship between length and genomic offset (A–C) or environmental distance (D) including disease in the model. Each point represents mean length in a different bag. Lewisetta is shown in green, whereas York River is shown in blue. Kendall's Tau for each site is shown in boxes. Significant correlations are indicated with a star symbol and represented with a solid line, whereas non‐significant correlations use dashed lines. Note that these graphs represent analyses conducted without the selection lines S1‐LOLA and S2‐DEBY.

Here we present correlations between genomic offset models, built with disease, and two fitness proxies, survival and length. Within a common garden, a genomic offset model that performs well should have a significant negative correlation between predicted offset and empirical mean fitness proxy across populations (Lotterhos 2024b). All genomic offset methods showed a negative trend between offset and both survival and length, though the correlation between survival and offset was only significant for LEA_offset_ at Lewisetta (Lewisetta, *τ*_LEA_ = −0.546) (Figure 4). When selection lines S1‐LOLA and S2‐DEBY were removed from the correlation analysis, correlations between survival and genomic offset were generally weaker and were never significant (Table S2). Length was better predicted by genomic offset, with a significant negative correlation between this fitness proxy and all methods at both sites, except RDA_offset_ at York River (Lewisetta, *τ*_LEA_ = −0.660; *τ*_RDA_ = −0.508; *τ*_GF_ = −0.447; York River, *τ*_LEA_ = −0.457; *τ*_GF_ = −0.494) (Figure 5). Again, correlations between length and offset without selection lines were weaker and only significant for LEA_offset_ and RDA_offset_ at Lewisetta (Table S2). Differences in predictions across methods, described above, expectedly propagated to differences in their predictive performance, with LEA_offset_ and RDA_offset_ generally having the strongest negative correlations with both survival and length (Figures 4 and 5). Environmental distance had a negative association with both fitness proxies at Lewisetta, but was positively correlated with length at York River, and was only significantly correlated with length at Lewisetta. The correlation between environmental distance and survival was never significant, but was generally on par with genomic offsets.

Across common gardens, a genomic offset method that performs well should have higher offsets to gardens where fitness is lower, and lower offsets to gardens where fitness is higher. In other words, across common gardens, the magnitude of differences in offsets should match the magnitude of differences in fitness. Here we found a low magnitude of differences in offset between common gardens (GF_offset_ 17.6%, LEA_offset_ 17.2%, RDA_offset_ 5.2% difference between Lewisetta and York River), but large differences in fitness proxies (survival 56.8%, length 27.3% difference between Lewisetta and York River).

### 3.5 Effect of the Biotic Landscape on Genomic Offset Prediction and Performance

Genomic offset predictions differed depending on the environmental variable set used for training and prediction, but not in a consistent way. For survival at the Lewisetta site, predicted offset was more negatively correlated with survival at Lewisetta upon inclusion of disease in the genomic offset prediction (|∆*τ*| = 0.110 average stronger correlation across methods comparing models built with disease + abiotic variables to models built only with abiotic variables). In contrast, at the York River site, models that included disease variables in offset prediction had no consistent impact on genomic offset method performance compared to offset models built only on abiotic variables and weakened the strength of correlation between RDA_offset_ and survival. This is counterintuitive because the York River site had higher disease pressure, so we would have expected that the inclusion of disease variables would have improved performance at this site. It is important to note that all correlations between genomic offset and survival were statistically insignificant, regardless of whether disease variables were included.

Inclusion of the disease landscape also consistently improved offset correlation with length at Lewisetta (|∆*τ*| = 0.108 stronger correlation) but had a negligible and inconsistent impact at York River. Nearly all correlations between length and disease + abiotic offset were significant, with the exception of RDA_offset_ at York River, whereas the correlation between length and abiotic RDA_offset_ was not significant at either site. Environmental distance was significantly negatively correlated with length at Lewisetta both with and without the inclusion of disease, but never at York River. When selection lines DEBY and LOLA were removed from analysis, the inclusion of disease led to larger improvements in correlation between offset and both fitness proxies than when these groups were included, though we reiterate that correlations were still overall weaker when selection lines were removed (Table 3, Table S2).

**TABLE 3:** Experimental fitness correlation with genomic offset, with and without disease.

Method	Common garden	*τ*_survival, no disease_	*τ*_survival, disease_	∆*τ*_survival_	*τ*_length, no disease_	*τ*_length, disease_	∆*τ*_length_
				Disease—no disease model (+ is improvement)			Disease—no disease model (+ is improvement)
EnvDist	Lewisetta	−0.137	−0.205	+0.068	−0.447 *	−0.516 *	+0.069
LEA	Lewisetta	−0.137	−0.546 *	+0.409	−0.463 *	−0.660 ***	+0.197
RDA	Lewisetta	−0.053	−0.364	+0.311	−0.243	−0.508 *	+0.265
GF	Lewisetta	−0.068	−0.137	+0.069	−0.379 *	−0.447 *	+0.068
EnvDist	York River	−0.131	−0.077	−0.054	−0.303	−0.249	−0.054
LEA	York River	−0.231	−0.177	−0.054	−0.439 *	−0.457 *	+0.018
RDA	York River	−0.258	−0.367	+0.109	−0.195	−0.303	+0.108
GF	York River	−0.149	−0.285	+0.136	−0.485 *	−0.494 *	+0.009

*Note:* Correlations between genomic offset and survival (left) and length (right) at Lewisetta (top) and York River (bottom), with and without disease in the model. The improvement in performance of each method, given by the difference between the fitness proxy correlation with models built with disease + abiotic variables and models built only with abiotic variables, is shown as ∆*τ*. Positive change indicates that disease improved method performance, shown in green, whereas negative change indicates that disease worsened method performance, shown in red. Note that correlations were calculated including the selection lines S1‐LOLA and S2‐DEBY. Significance is indiciated by * *p* < 0.05, ** *p* < 0.01, *** *p* < 0.001.

## 4 Discussion

In this experiment, we found oysters had significantly lower survival and length at a moderate salinity, higher disease site (York River) compared to a low salinity, lower disease site (Lewisetta) (Figure 1E, Figure S1). Including disease in our offset models improved method performance to a greater degree at the site with lower disease pressure, which was unexpected. Although overall there were negative relationships among sites and fitness proxies, the slopes and significance of these relationships were dependent on the site of the test (with performance generally better at the lower disease site) and offset method being used. We also found a similar range of offset values to both sites despite the large difference in fitness proxies between the two sites. These results highlight issues with scaling up genomic offsets to multiple sites across an entire species range.

### 4.1 Drivers of Experimental Fitness Differences

Oyster survival and length were substantially lower at York River than at Lewisetta, suggesting that York River was impacted by some stressor(s) not present at Lewisetta. Lower salinity is associated with lower MSX ( *H. nelsoni* ) and Dermo ( *P. marinus* ) pressure (Mackin 1951; Andrews 1979; Ford and Haskin 1982; Ragone Calvo et al. 2003; Bushek et al. 2012). MSX was not detected at Lewisetta, our low salinity site, during the experimental period, and Dermo prevalence at York River, our moderate salinity site, was twice that of Lewisetta (Figure 1E, Figure S1). Pea crab (Zaops sp.) infections are also inhibited by low salinity, matching with our observation that approximately half of all oysters at York River were infected with pea crabs at the conclusion of the experiment, but no such infections were found at Lewisetta (Figure S1; O'Beirn and Walker 1999). Taken together, these results point to disease caused by marine parasite infection as a strong driver of fitness proxy differences between oysters deployed at different common garden sites.

Differences in population‐level survival and length averages within and across sites may also be explained by other factors including adaptive genetic differences resulting from differences in historic environmental regimes (Marshall et al. 2021; Wadgymar et al. 2022). Previous work has found evidence for such genetic‐environment interactions in this system, potentially leading to fitness consequences that vary across environmental gradients (Burford et al. 2014; Hughes et al. 2017; Hornick and Plough 2022; Bajaj et al. 2026). Oysters sourced from Louisiana (W2‐LA), which has no historical exposure to MSX, had substantially lower survival and shorter lengths at the MSX endemic York River site (Ewart and Ford 1993). Interestingly, despite similarly low historic exposure to MSX (Ewart and Ford 1993), oysters from Texas and Florida grouped with local populations and selection lines at both common garden sites. This may be due to effects of other environmental factors, such as exposure to hotter temperatures and higher Dermo pressure, similar to conditions at the common garden sites (Figure 1D,E; Bajaj et al. 2026). The Northern populations (W6‐NH, W6‐ME), in contrast, came from much cooler environments and have only been exposed to Dermo since the early 1990s (Mackin 1951; Ford and Haskin 1982; Ewart and Ford 1993; Burreson and Ford 2004), so may have struggled to cope with the warmer temperatures and higher disease pressure in the Chesapeake Bay (Figure 1D,E; Andrews 1979), although we cannot rule out other potential drivers of variation in population fitness without further study. The significant decline in Northern oysters' survival and length in hotter environments with increased disease prevalence suggests higher vulnerability to climate change in the Northern end of this species' range (Crespi 2000; Brady, Bolnick, Angert, et al. 2019; Brady, Bolnick, Barrett, et al. 2019).

### 4.2 Statistical Nuances of Genomic Offsets

Genomic offsets should have a negative relationship with experimental fitness proxies, as higher offset is interpreted to indicate more maladaptation to the future climate (i.e., lower fitness). Previous studies have used a number of metrics to look at genomic offset model performance, recovering correlations (Pearson, Spearman, Kendall) between −0.90 (good) and +0.50 (bad) and *R* ^2^ between 0.22 and 0.78 (Rhoné et al. 2020; Fitzpatrick et al. 2021; Láruson et al. 2022; Gain et al. 2023; Lachmuth et al. 2023, 2024; Lind et al. 2024; Archambeau et al. 2026; Lind and Lotterhos 2025; Verrico et al. 2025). We found similar variability in model performance across common gardens, as models generally performed worse at York River compared to Lewisetta. This discrepancy in performance across validation experiments may be caused by violations of the underlying assumptions of genomic offsets or by limitations of the methods themselves (Lotterhos 2024a; Ahrens et al. 2025).

Genomic offset methods are also limited by their training data. Little is known about the extent to which genomic offsets can be extrapolated to populations or environments beyond their training dataset (Lotterhos 2024b). Recent simulation work has shown that extrapolating to environments beyond the known genetic‐environment relationship reduces offset performance, especially in systems where local adaptation is strong (Lind and Lotterhos 2025). Here, neither the common garden environments nor the experimental source population sites‐of‐origin fell outside the range of seascape training data, so this cannot explain poor model performance. It is also critical to identify relevant drivers of fitness, as including nonsense variables or disincluding relevant environmental variables can both obscure the true selective landscape and worsen model predictions (Rellstab et al. 2015; Riginos et al. 2016; Dauphin et al. 2023). In addition, inconsistencies in the genotype–phenotype map across divergent populations would degrade consistency in the genotype‐environment signal that genomic offset models rely on, undermining their predictive accuracy (Johnson et al. 2023; Rêgo et al. 2025).

Here, incorporating disease, a critical driver of differential survival in this system, in addition to abiotic variables into genomic offset models improved the performance of most methods at the low disease site, but less so at the high disease site. There are different possible reasons for this counter‐intuitive result. On one hand, stronger selection at the more stressful high disease site could amplify epistatic interactions among populations of different genetic and environmental backgrounds (de Vos et al. 2013; Gupta and Adami 2016), which would lead to lower accuracy of genomic offsets at sites with stronger selection. Our training dataset also has significant limitations for the disease data, namely a lack of temporal resolution for the 33 seascape sites and experimental source population sites‐of‐origin, and a consequent inability to account for differential disease dynamics and seasonality in Dermo and MSX. Inaccurate estimates of disease in the training data may introduce noise into the genotype‐environment association for disease, but this does not explain why incorporating disease would result in a performance improvement at some sites and not others. Although acknowledging these limitations to our disease dataset, we note that offset models historically do not consider disease at all, which could worsen predictions in systems with significant disease pressure (Dauphin et al. 2023). Future work should further explore the extent to which environmental novelty and predictor set impact offset predictions and correlation with fitness proxy traits, particularly in systems with strong biotic selective pressures (Lotterhos 2024b).

Another critical assumption is that populations are locally adapted and currently reside in their adaptive optimum (Fitzpatrick and Keller 2015; Rellstab et al. 2016; Capblancq et al. 2020; Gain et al. 2023). Local adaptation is common in nature, having long been documented in terrestrial species and increasingly in marine species (Conover et al. 2006; Leimu and Fischer 2008; Hereford 2009; Sanford and Kelly 2011). Despite this, it is difficult to definitively prove local adaptation, and many systems show other patterns of variation across environments such as countergradient variation or maladaptation (Crespi 2000; Conover and Schultz 1995; Brady, Bolnick, Angert, et al. 2019; Browne et al. 2019; Anderson and Wadgymar 2020; Albecker et al. 2025). In systems where local adaptation is weak or absent, genomic offsets may inaccurately infer patterns of adaptive variation and thus be poor predictors of fitness (Lind and Lotterhos 2025). Although eastern oysters have documented responses to changes in salinity, temperature and disease, reciprocal transplant experiments find mixed support for local adaptation (Burford et al. 2014; Hughes et al. 2017). Given that non‐local Southern oysters often outperformed local Virginia oysters and selection lines, local adaptation may be limited in this system, violating this fundamental assumption of genomic offset methods. Although we cannot rule out lack of local adaptation in our system as a cause for poor performance at one common garden site, our genomic offset models' failure to capture large differences in fitness proxies between the two sites illustrates a potentially larger issue within our results.

### 4.3 Implications for Applications of Genomic Offsets

Genomic offsets were originally proposed as an estimate of maladaptation to future climate change (Fitzpatrick and Keller 2015), but have recently gained momentum for other applications in conservation, population and fisheries management, aquaculture and agriculture—each with different goals for offset predictions (Bay et al. 2018; Rellstab et al. 2021; Jacquemart et al. 2025; Beck et al. 2025). Conservation actions such as restoration could use genomic offsets to identify populations best suited to environmental conditions at a specific location (an application that compares the fitness of different genotypes at a single environment), whereas other actions could benefit from knowing the degree to which different populations within a species will be vulnerable to climate change (an application that compares the fitness of a single genotype in different environments) (Lotterhos 2024a). For the latter case, stakeholders are generally concerned with identifying populations that will either decline (maladapted) or be robust (pre‐adapted) to climate change for subsequent interventions like assisted migration or seed banking (Capblancq et al. 2020; Rellstab et al. 2021; Gougherty et al. 2021; Chen et al. 2022; Varas‐Myrik et al. 2024). In agriculture and aquaculture, genomic offsets are being proposed as a solution to account for complex genetic–environment effects in selective breeding programs seeking to maximize desirable traits such as survival, growth and yield under climate change (Goddard and Hayes 2009; Cole et al. 2021; Feng et al. 2024; Beck et al. 2025). In order to make ecologically meaningful predictions of maladaptation or to be incorporated into modern breeding strategies and algorithms, however, offset values must consistently translate to specific fitness values or biologically relevant thresholds (Lotterhos 2024a).

**FIGURE 6:**
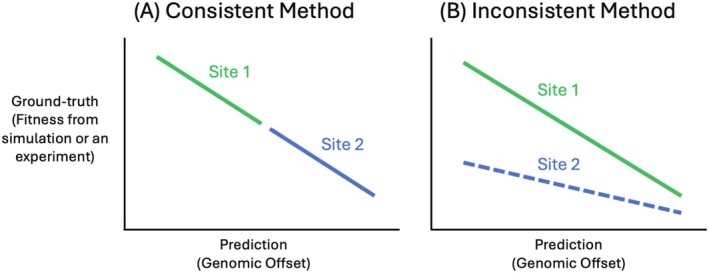
Conceptual diagram depicting different offset‐fitness relationships across common garden test sites. (A) Strong offset‐fitness relationship: The slope and intercept of the offset‐fitness relationship are maintained across sites, indicating that the magnitude of offset reliably predicts the magnitude of fitness loss across space. (B) Weak offset‐fitness relationship: The strength of the offset‐fitness relationship varies across sites, and the magnitude of offsets does not translate to the same fitness proxy value across space.

For applications seeking to estimate a maladaptation of a population or genotype in one environment to another environment (either spatially from one location to another or temporally from the present to the future), fitness traits and offsets must be clearly linked across methodologies and in different environmental contexts, and there should be consistency in the magnitude of offset differences and absolute fitness differences in the dataset (Lotterhos 2024b). Specifically, the relationship between the genomic offset and a fitness proxy has to be preserved across different sites in a species range (Figure 6A). For instance, sites in which genotypes will be less maladapted to climate change have lower genomic offsets (green site in Figure 6A, analogous to Lewisetta in our study), whereas sites in which genotypes will be more maladapted to climate change have higher genomic offsets (blue site in Figure 6A, analogous to York River in our study). If both the slope and the intercept of the offset‐fitness relationship are not preserved across sites (as shown in Figure 6B), range‐wide forecasts of maladaptation to climate change will be inaccurate.

In contrast, for applications seeking to predict the relative fitness of one genotype vs. another at a specific common garden site (e.g., restoration), the relationship in Figure 6 Left does not have to be preserved across sites. In this case, validating the offset‐fitness relationship at the specific site is sufficient for the offsets to be used in application (such as Site 1 in Figure 6B). For small scale restoration projects, where the goal is to identify one or a few ideal donor populations at a specific site, genomic offsets may be a useful tool to rank potential source populations by their relative suitability to site conditions (Houde et al. 2015; Rellstab et al. 2021). However, offsets may work inconsistently across restoration sites, as was the case here, and incorrectly rank population fitness. For example, most methods predicted New Hampshire to have relatively low offset to York River, but in reality it fared significantly worse than other populations, making it a poor choice to move to this site.

Only a handful of studies have evaluated offsets in multiple common gardens, and of those even fewer explicitly show the fitness‐offset relationship across sites (e.g., Figure 6). Previous validation experiments generally find a negative relationship across gardens, but different studies recover variable strength and explanatory power, especially depending on the choice of fitness proxy (Lachmuth et al. 2023; Lind et al. 2024; Verrico et al. 2025). For example, common garden evaluations linking red spruce height‐growth to offset have recovered relatively consistent fitness‐offset relationships across gardens, but this correlation weakens for other fitness traits (Lachmuth et al. 2023; Verrico et al. 2025). Fitness is broadly defined as the ability of organisms to survive and reproduce (Barker 2009; Orr 2009; Wadgymar et al. 2024). When direct measurements of survival and reproduction are difficult to obtain, fitness is sometimes approximated by size, as larger organisms are assumed to have higher longevity and fecundity (e.g., Rundle et al. 2003; Rollins 2017; Ahti et al. 2020). Though system‐specific limitations, namely population‐specific differences in the timing of gonadal development and broadcast spawning, precluded us from collecting useful reproductive output data, weak correlations between survival and offset indicate that at least one of these more direct fitness components is poorly predicted by genomic offset. Although we found that offset correlations with length are much stronger than with survival, especially at Lewisetta, in line with previously observed relationships between growth and offset, we emphasize that length is a less relevant fitness proxy than survival (Verrico et al. 2025). Importantly, previous studies also did not encounter large site‐level differences in fitness‐proxy averages like those observed here, so they would not display inconsistency between offset and fitness‐proxy values. We found that the exact same value of genomic offset translated to at times over 50% difference in survival (Figures 3 and 5b), raising issues about directly translating genomic offsets into a metric of population vulnerability. Furthermore, we found that the correlations between offset and fitness proxies were inconsistent across sites even across a relatively small geographical range, pointing to issues with scaling offsets across entire species ranges.

These issues highlight the importance of ground‐truthing offset predictions with other lines of evidence (e.g., observations of performance or fitness in the field) and checking that the relationship is consistent across multiple common gardens. These issues could potentially be addressed through hybrid modelling approaches that incorporate species abundance or demographic data such as ecological niche, species distribution or demographic models (Bay et al. 2018; Chen et al. 2022). In systems where genomic offsets are validated, they could be a powerful predictive tool, but should still be interpreted cautiously when scaled across large geographic ranges or being used to interpret population vulnerability to climate change.

## 5 Conclusion

Here we used common garden experiments to understand how the genetic and environmental histories of the eastern oyster interact to determine outcomes across a salinity and disease gradient. We then pair these results with genomic offsets to understand whether and how offset methods can be used to make meaningful predictions about fitness changes across the seascape. We found that Southern populations, mid‐Atlantic populations, and selection lines perform well at common gardens, whereas Northern oyster populations suffer substantial mortality when moved south to the Chesapeake Bay. Though relationships between genomic offset and experimental fitness proxies were negative, these correlations were often weak and insignificant, with vast differences in model performance between common garden sites. Discrepancies between predicted offset and empirical fitness proxies suggest that genomic offsets were not sufficiently accurate to be useful in this system, particularly when scaling across entire species ranges to assess vulnerability to climate. Genomic offset methods require further validation across a multitude of systems to determine when and if they can be reliably applied in real‐world settings.

## Author Contributions

**Camille A. Rumberger:** methodology, validation, formal analysis, investigation, data curation, writing – original draft, visualization, supervision, project administration. **Madeline G. Eppley:** investigation, data curation. **Kiran Bajaj:** investigation, data curation. **Nicole Mongillo:** investigation, data curation. **Shelley Katsuki:** investigation, project administration. **Jessica A. Small:** conceptualization, methodology, investigation, resources, project administration. **Katie E. Lotterhos:** conceptualization, methodology, investigation, resources, data curation, supervision, project administration. All authors contributed to review and editing of the manuscript.

## Funding

This work was supported by the National Science Foundation (2043905).

## Conflicts of Interest

The authors declare no conflicts of interest.

## Supporting information


**File S1:** Experimental oyster survival.
**File S2:** Experimental oyster lengths.
**File S3:** Offsets from experimental populations to common gardens.
**File S4:** Offsets from seascape populations to common gardens.
**Figure S1:** Disease pressure during the experimental period at both common garden sites. (A) MSX prevalence through time. (B) Dermo prevalence through time. (C) Median prevalence of MSX, Dermo and pea crabs during the common garden experiment.
**Figure S2:** (A) Salinity, measured in parts per thousand, and (B) temperature, measured in degrees Celsius, at common garden sites over the experimental period.
**Figure S3:** Environmental distances between source population sites‐of‐origin and common gardens, calculated as Euclidean distance.
**Figure S4:** Pairwise *F*_ST_ of wild experimental source populations.
**Figure S5:** Fitness‐Proxies throughout the common garden experiment. Each data point represents population level survival or length averages at each monitoring event.
**Figure S6:** Correlations between predicted offset and experimental survival thru time. LEA_offset_, RDA_offset_, GF_offset_ and environmental distance are all shown.
**Figure S7:** Correlations between predicted offset and experimental length through time. LEA_offset_, RDA_offset_, GF_offset_ and environmental distance are all shown.
**Figure S8:** Correlations between offset predicted by different methods. Comparisons are between LEA_offset_, RDA_offset_, GF_offset_ and environmental distance at each common garden site. Correlations reported are Kendall's Tau.
**Table S1:** Geographic information about populations included in the seascape training dataset.
**Table S2:** Population genetic statistics of the six wild experimental source populations, including expected (*H*_e_) and observed (*H*_o_) heterozygosity, as well as inbreeding coefficient (*F*_IS_).
**Table S3:** Experimental fitness correlation with genomic offset, with and without disease, calculated without inclusion of selection lines.

## Data Availability

Data are archived on BCO‐DMO in the project ‘CAREER: Evaluation of machine learning algorithms for understanding and predicting adaptation to multivariate environments with a Model Validation Program (MVP)’ available at https://www.bco‐dmo.org/project/876610. Code is archived on Zenodo at the following DOI: http://doi.org/10.5281/zenodo.20331286.

## References

[mec70457-bib-0001] Ahrens, C. W. , P. D. Rymer , and A. D. Miller . 2025. “Genetic Offset and Vulnerability Modelling Under Climate Change Scenarios: Common Misinterpretations and Violations of Evolutionary Principles.” Evolution 80, no. 1: 15–27. 10.1093/evolut/qpaf216 41092287

[mec70457-bib-0002] Ahti, P. A. , A. Kuparinen , and S. Uusi‐Heikkilä . 2020. “Size Does Matter—The Eco‐Evolutionary Effects of Changing Body Size in Fish.” Environmental Reviews 28, no. 3: 311–324.

[mec70457-bib-0003] Albecker, M. A. , T. B. Bittar , G. C. Trussell , and K. E. Lotterhos . 2025. “A Quantitative Survey of Cogradient and Countergradient Variation in Nature.” American Naturalist 206, no. 5: 385–402. 10.1086/737682 41172332

[mec70457-bib-0004] Anderson, J. T. , and S. M. Wadgymar . 2020. “Climate Change Disrupts Local Adaptation and Favours Upslope Migration.” Ecology Letters 23, no. 1: 181–192. 31729141 10.1111/ele.13427

[mec70457-bib-0005] Andrews, J. 1979. “Oyster Diseases in Chesapeake Bay.” Journal of Marine Science 41: 45.

[mec70457-bib-0006] Archambeau, J. , M. Benito Garzón , M. de Miguel , et al. 2026. “Evaluating Genomic Offset Predictions in a Forest Tree With High Population Genetic Structure.” American Naturalist 207, no. 3: 389–414. 10.1086/739045 41730226

[mec70457-bib-0007] Bajaj, K. , N. Mongillo , M. G. Eppley , et al. 2026. “Contrasting Effects of Geographic Distance, Environmental Distance, and Intraspecific Diversity on the Performance of Eastern Oysters ( *Crassostrea virginica* ) in Common Gardens.” *bioRxiv* 2026.04.02.716183. 10.64898/2026.04.02.716183v1.

[mec70457-bib-0008] Barker, J. S. F. 2009. “Defining Fitness in Natural and Domesticated Populations.” In Adaptation and Fitness in Animal Populations, 3–14. Springer Netherlands.

[mec70457-bib-0009] Bates, D. , M. Mächler , B. Bolker , and S. Walker . 2015. “Fitting Linear Mixed‐Effects Models Usinglme4.” Journal of Statistical Software 67, no. 1: 1–48.

[mec70457-bib-0010] Bay, R. A. , R. J. Harrigan , V. L. Underwood , H. L. Gibbs , T. B. Smith , and K. Ruegg . 2018. “Genomic Signals of Selection Predict Climate‐Driven Population Declines in a Migratory Bird.” Science (New York, N.Y.) 359, no. 6371: 83–86. 29302012 10.1126/science.aan4380

[mec70457-bib-0011] Beck, S. V. , S. A. May , T. Kess , I. R. Bradbury , E. A. Lozada‐Soto , and M. Wellenreuther . 2025. “Applying Genomic Offsets to Breeding Programmes: Bridging Evolutionary Insights With Practical Applications.” Evolutionary Applications 18, no. 10: e70155. 41158441 10.1111/eva.70155 PMC12557460

[mec70457-bib-0012] Benjamini, Y. , and Y. Hochberg . 1995. “Controlling the False Discovery Rate: A Practical and Powerful Approach to Multiple Testing.” Journal of the Royal Statistical Society. Series B, Statistical Methodology 57, no. 1: 289–300.

[mec70457-bib-0013] Brady, S. P. , D. I. Bolnick , A. L. Angert , et al. 2019. “Causes of Maladaptation.” Evolutionary Applications 12, no. 7: 1229–1242. 31417611 10.1111/eva.12844 PMC6691215

[mec70457-bib-0014] Brady, S. P. , D. I. Bolnick , R. D. H. Barrett , et al. 2019. “Understanding Maladaptation by Uniting Ecological and Evolutionary Perspectives.” American Naturalist 194, no. 4: 495–515. 10.1086/705020 31490718

[mec70457-bib-0015] Browne, L. , J. W. Wright , S. Fitz‐Gibbon , P. F. Gugger , and V. L. Sork . 2019. “Adaptational Lag to Temperature in Valley Oak ( *Quercus lobata* ) Can Be Mitigated by Genome‐Informed Assisted Gene Flow.” Proceedings of the National Academy of Sciences of the United States of America 116, no. 50: 25179–25185. 31767740 10.1073/pnas.1908771116 PMC6911187

[mec70457-bib-0016] Burford, M. O. , J. Scarpa , B. J. Cook , and M. P. Hare . 2014. “Local Adaptation of a Marine Invertebrate With a High Dispersal Potential: Evidence From a Reciprocal Transplant Experiment of the Eastern Oyster *Crassostrea virginica* .” Marine Ecology Progress Series 505: 161–175.

[mec70457-bib-0017] Burge, C. A. , C. Mark Eakin , C. S. Friedman , et al. 2014. “Climate Change Influences on Marine Infectious Diseases: Implications for Management and Society.” Annual Review of Marine Science 6, no. 1: 249–277. 10.1146/annurev-marine-010213-135029 23808894

[mec70457-bib-0018] Burreson, E. M. , and S. E. Ford . 2004. “A Review of Recent Information on the Haplosporidia, With Special Reference to *Haplosporidium nelsoni* (MSX Disease).” Aquatic Living Resources 17, no. 4: 499–517.

[mec70457-bib-0019] Bushek, D. , S. Ford , and I. Burt . 2012. “Long‐Term Patterns of an Estuarine Pathogen Along a Salinity Gradient.” Journal of Marine Research 70, no. 2: 225–251.

[mec70457-bib-0020] Capblancq, T. , M. C. Fitzpatrick , R. A. Bay , M. Exposito‐Alonso , and S. R. Keller . 2020. “Genomic Prediction of (Mal)adaptation Across Current and Future Climatic Landscapes.” Annual Review of Ecology, Evolution, and Systematics 51, no. 1: 245–269.

[mec70457-bib-0021] Capblancq, T. , and B. R. Forester . 2021. “Redundancy Analysis: A Swiss Army Knife for Landscape Genomics.” Methods in Ecology and Evolution 12, no. 12: 2298–2309.

[mec70457-bib-0022] Capblancq, T. , S. Lachmuth , M. C. Fitzpatrick , and S. R. Keller . 2023. “From Common Gardens to Candidate Genes: Exploring Local Adaptation to Climate in Red Spruce.” New Phytologist 237, no. 5: 1590–1605. 36068997 10.1111/nph.18465 PMC10092705

[mec70457-bib-0023] Carnegie, R. B. , and E. M. Burreson . 2011. “Declining Impact of an Introduced Pathogen: *Haplosporidium nelsoni* in the Oyster *Crassostrea virginica* in Chesapeake Bay.” Marine Ecology Progress Series 432: 1–15.

[mec70457-bib-0024] Chen, Y. , Z. Jiang , P. Fan , et al. 2022. “The Combination of Genomic Offset and Niche Modelling Provides Insights Into Climate Change‐Driven Vulnerability.” Nature Communications 13, no. 1: 4821. 10.1038/s41467-022-32546-z PMC9381542 35974023

[mec70457-bib-0025] Cole, J. B. , J. W. Dürr , and E. L. Nicolazzi . 2021. “Invited Review: The Future of Selection Decisions and Breeding Programs: What Are We Breeding for, and Who Decides?” Journal of Dairy Science 104, no. 5: 5111–5124. 33714581 10.3168/jds.2020-19777

[mec70457-bib-0026] Conover, D. O. , L. M. Clarke , S. B. Munch , and G. N. Wagner . 2006. “Spatial and Temporal Scales of Adaptive Divergence in Marine Fishes and the Implications for Conservation.” Journal of Fish Biology 69: 21–47.

[mec70457-bib-0027] Conover, D. O. , and E. T. Schultz . 1995. “Phenotypic Similarity and the Evolutionary Significance of Countergradient Variation.” Trends in Ecology & Evolution 10, no. 6: 248–252. 21237029 10.1016/S0169-5347(00)89081-3

[mec70457-bib-0028] Crespi, B. J. 2000. “The Evolution of Maladaptation.” Heredity 84, no. Pt 6: 623–629. 10886377 10.1046/j.1365-2540.2000.00746.x

[mec70457-bib-0029] Dauphin, B. , C. Rellstab , R. O. Wüest , et al. 2023. “Re‐Thinking the Environment in Landscape Genomics.” Trends in Ecology & Evolution 38, no. 3: 261–274. 36402651 10.1016/j.tree.2022.10.010

[mec70457-bib-0030] Dawson, T. P. , S. T. Jackson , J. I. House , I. C. Prentice , and G. M. Mace . 2011. “Beyond Predictions: Biodiversity Conservation in a Changing Climate.” Science (New York, N.Y.) 332, no. 6025: 53–58. 21454781 10.1126/science.1200303

[mec70457-bib-0031] de Vos, M. G. J. , F. J. Poelwijk , N. Battich , J. D. T. Ndika , and S. J. Tans . 2013. “Environmental Dependence of Genetic Constraint.” PLoS Genetics 9, no. 6: e1003580. 23825963 10.1371/journal.pgen.1003580 PMC3694820

[mec70457-bib-0032] Ding, H. , and A. J. Elmore . 2015. “Spatio‐Temporal Patterns in Water Surface Temperature From Landsat Time Series Data in the Chesapeake Bay, U.S.A.” Remote Sensing of Environment 168: 335–348.

[mec70457-bib-0033] Dungan, C. F. , and D. Bushek . 2015. “Development and Applications of Ray's Fluid Thioglycollate Media for Detection and Manipulation of Perkinsus spp. Pathogens of Marine Molluscs.” Journal of Invertebrate Pathology 131: 68–82. 26003823 10.1016/j.jip.2015.05.004

[mec70457-bib-0034] Eppley, M. G. , K. Bajaj , C. Rumberger , et al. 2026. “Range‐Wide Genetic Population Structure and Environmental Adaptation in the Eastern Oyster ( *Crassostrea virginica* ) Provides Insight for Aquaculture.” *bioRxiv* 2026.03.30.715280. 10.64898/2026.03.30.715280. PMC13446745 42561033

[mec70457-bib-0035] Ewart, J. W. , and S. E. Ford . 1993. History and Impact of MSX and Dermo Diseases on Oyster Stocks in the Northeast Region (NRAC Fact Sheet 200). Northeastern Regional Aquaculture Center.

[mec70457-bib-0036] Feng, J. , X. Dan , Y. Cui , et al. 2024. “Integrating Evolutionary Genomics of Forest Trees to Inform Future Tree Breeding Amid Rapid Climate Change.” Plant Communications 5, no. 10: 101044. 39095989 10.1016/j.xplc.2024.101044 PMC11573912

[mec70457-bib-0038] Fitzpatrick, M. C. , V. E. Chhatre , R. Y. Soolanayakanahally , and S. R. Keller . 2021. “Experimental Support for Genomic Prediction of Climate Maladaptation Using the Machine Learning Approach Gradient Forests.” Molecular Ecology Resources 21, no. 8: 2749–2765. 33683822 10.1111/1755-0998.13374

[mec70457-bib-0039] Fitzpatrick, M. C. , and S. R. Keller . 2015. “Ecological Genomics Meets Community‐Level Modelling of Biodiversity: Mapping the Genomic Landscape of Current and Future Environmental Adaptation.” Ecology Letters 18, no. 1: 1–16. 25270536 10.1111/ele.12376

[mec70457-bib-0040] Fitzpatrick, M. C. , S. R. Keller , and K. E. Lotterhos . 2025. “The Challenge of Genomic Forecasting in an Era of Global Change.” American Naturalist 207, no. 3: 738891. 10.1086/738891 41730225

[mec70457-bib-0041] Ford, S. E. , and H. H. Haskin . 1982. “History and Epizootiology of *Haplosporidium nelsoni* (MSX), an Oyster Pathogen in Delaware Bay, 1957–1980.” Journal of Invertebrate Pathology 40, no. 1: 118–141.

[mec70457-bib-0042] Frank‐Lawale, A. , S. K. Allen Jr. , and L. Dégremont . 2014. “Breeding and Domestication of Eastern Oyster ( *Crassostrea virginica* ) Lines for Culture in the Mid‐Atlantic, USA: Line Development and Mass Selection for Disease Resistance.” Journal of Shellfish Research 33, no. 1: 153–165.

[mec70457-bib-0043] Frichot, E. , and O. François . 2015. “LEA: An R Package for Landscape and Ecological Association Studies.” Methods in Ecology and Evolution 6, no. 8: 925–929.

[mec70457-bib-0044] Gain, C. , and O. François . 2021. “LEA 3: Factor Models in Population Genetics and Ecological Genomics With R.” Molecular Ecology Resources 21, no. 8: 2738–2748. 33638893 10.1111/1755-0998.13366

[mec70457-bib-0045] Gain, C. , B. Rhoné , P. Cubry , et al. 2023. “A Quantitative Theory for Genomic Offset Statistics.” Molecular Biology and Evolution 40, no. 6: msad140. 10.1093/molbev/msad140. 37307566 PMC10306404

[mec70457-bib-0046] Goddard, M. E. , and B. J. Hayes . 2009. “Mapping Genes for Complex Traits in Domestic Animals and Their Use in Breeding Programmes.” Nature Reviews Genetics 10, no. 6: 381–391. 10.1038/nrg2575 19448663

[mec70457-bib-0047] Goudet, J. , and T. Jombart . 2024. “hierfstat: Estimation and Tests of Hierarchical *F*‐Statistics.” R Package Version 0.5‐11. https://github.com/jgx65/hierfstat.

[mec70457-bib-0048] Gougherty, A. V. , S. R. Keller , and M. C. Fitzpatrick . 2021. “Maladaptation, Migration and Extirpation Fuel Climate Change Risk in a Forest Tree Species.” Nature Climate Change 11, no. 2: 166–171.

[mec70457-bib-0049] Grabowski, J. H. , R. D. Brumbaugh , R. F. Conrad , et al. 2012. “Economic Valuation of Ecosystem Services Provided by Oyster Reefs.” Bioscience 62, no. 10: 900–909.

[mec70457-bib-0050] Gräler, B. , E. Pebesma , and G. Heuvelink . 2016. “Spatio‐Temporal Interpolation Using gstat.” R Journal 8, no. 1: 204.

[mec70457-bib-0051] Griffiths, J. S. , K. M. Johnson , and M. W. Kelly . 2021. “Evolutionary Change in the Eastern Oyster, *Crassostrea virginica* , Following Low Salinity Exposure.” Integrative and Comparative Biology 61, no. 5: 1730–1740. 34448845 10.1093/icb/icab185

[mec70457-bib-0052] Gupta, A. , and C. Adami . 2016. “Strong Selection Significantly Increases Epistatic Interactions in the Long‐Term Evolution of a Protein.” PLoS Genetics 12, no. 3: e1005960. 27028897 10.1371/journal.pgen.1005960 PMC4814079

[mec70457-bib-0053] Halekoh, U. , and S. Højsgaard . 2014. “A Kenward–Roger Approximation and Parametric Bootstrap Methods for Tests in Linear Mixed Models—The R Package Pbkrtest.” Journal of Statistical Software 59, no. 9: 1–32.26917999

[mec70457-bib-0054] Hereford, J. 2009. “A Quantitative Survey of Local Adaptation and Fitness Trade‐Offs.” American Naturalist 173, no. 5: 579–588. 10.1086/597611 19272016

[mec70457-bib-0055] Hornick, K. M. , and L. V. Plough . 2022. “Genome‐Wide Analysis of Natural and Restored Eastern Oyster Populations Reveals Local Adaptation and Positive Impacts of Planting Frequency and Broodstock Number.” Evolutionary Applications 15, no. 1: 40–59. 35126647 10.1111/eva.13322 PMC8792482

[mec70457-bib-0056] Houde, A. L. S. , S. R. Garner , and B. D. Neff . 2015. “Restoring Species Through Reintroductions: Strategies for Source Population Selection: Strategies for Source Population Selection.” Restoration Ecology 23, no. 6: 746–753.

[mec70457-bib-0057] Hughes, A. R. , T. C. Hanley , J. E. Byers , et al. 2017. “Genetic by Environmental Variation but no Local Adaptation in Oysters ( *Crassostrea virginica* ).” Ecology and Evolution 7, no. 2: 697–709. 28116064 10.1002/ece3.2614 PMC5243187

[mec70457-bib-0058] Jackson, D. A. 1993. “Stopping Rules in Principal Components Analysis: A Comparison of Heuristical and Statistical Approaches.” Ecology 74, no. 8: 2204–2214.

[mec70457-bib-0059] Jacquemart, A. S. , A. Tigano , M. K. Gale , et al. 2025. “Application of Genomic Offsets to Inform Freshwater Fisheries Management Under Climate Change.” Evolutionary Applications 18, no. 8: e70149. 40881941 10.1111/eva.70149 PMC12382357

[mec70457-bib-0060] Johnson, M. S. , G. Reddy , and M. M. Desai . 2023. “Epistasis and Evolution: Recent Advances and an Outlook for Prediction.” BMC Biology 21, no. 1: 120. 37226182 10.1186/s12915-023-01585-3 PMC10206586

[mec70457-bib-0061] Kachmar, M. L. , C. Bergman , H. J. Schreier , et al. 2025. “Spatio‐Temporal Patterns of *Perkinsus marinus* Infections Are Driven by a Changing Environment in the Chesapeake Bay.” Diseases of Aquatic Organisms 164: 111–127. 41263289 10.3354/dao03876

[mec70457-bib-0062] Kenward, M. G. , and J. H. Roger . 1997. “Small Sample Inference for Fixed Effects From Restricted Maximum Likelihood.” Biometrics 53, no. 3: 983–997. 9333350

[mec70457-bib-0064] Lachmuth, S. , T. Capblancq , S. R. Keller , and M. C. Fitzpatrick . 2023. “Assessing Uncertainty in Genomic Offset Forecasts From Landscape Genomic Models (and Implications for Restoration and Assisted Migration).” Frontiers in Ecology and Evolution 11: 1155783. 10.3389/fevo.2023.1155783.

[mec70457-bib-0065] Lachmuth, S. , T. Capblancq , A. Prakash , S. R. Keller , and M. C. Fitzpatrick . 2024. “Novel Genomic Offset Metrics Integrate Local Adaptation Into Habitat Suitability Forecasts and Inform Assisted Migration.” Ecological Monographs 94, no. 1: e1593.

[mec70457-bib-0067] Láruson, Á. J. , M. C. Fitzpatrick , S. R. Keller , B. C. Haller , and K. E. Lotterhos . 2022. “Seeing the Forest for the Trees: Assessing Genetic Offset Predictions From Gradient Forest.” Evolutionary Applications 15, no. 3: 403–416. 35386401 10.1111/eva.13354 PMC8965365

[mec70457-bib-0068] Layton, K. K. S. , M. S. O. Brieuc , R. Castilho , et al. 2024. “Predicting the Future of Our Oceans‐Evaluating Genomic Forecasting Approaches in Marine Species.” Global Change Biology 30, no. 3: e17236. 38519845 10.1111/gcb.17236

[mec70457-bib-0069] Leimu, R. , and M. Fischer . 2008. “A Meta‐Analysis of Local Adaptation in Plants.” PLoS One 3, no. 12: e4010. 19104660 10.1371/journal.pone.0004010 PMC2602971

[mec70457-bib-0070] Lind, B. M. , R. Candido‐Ribeiro , P. Singh , et al. 2024. “How Useful Is Genomic Data for Predicting Maladaptation to Future Climate?” Global Change Biology 30, no. 4: e17227. 38558300 10.1111/gcb.17227

[mec70457-bib-0071] Lind, B. M. , and K. E. Lotterhos . 2025. “The Accuracy of Predicting Maladaptation to New Environments With Genomic Data.” Molecular Ecology Resources 25, no. 4: e14008. 39212146 10.1111/1755-0998.14008 PMC11969643

[mec70457-bib-0072] Lotterhos, K. E. 2024a. “Interpretation Issues With ‘Genomic Vulnerability’ Arise From Conceptual Issues in Local Adaptation and Maladaptation.” Evolution Letters 8, no. 3: 331–339. 38818416 10.1093/evlett/qrae004 PMC11134465

[mec70457-bib-0073] Lotterhos, K. E. 2024b. “Principles in Experimental Design for Evaluating Genomic Forecasts.” Methods in Ecology and Evolution 15, no. 9: 1466–1482.

[mec70457-bib-0074] Mackin, J. G. 1951. “Histopathology of Infection of *Crassostrea virginica* (Gmelin) by *Dermocystidium marinum* Mackin, Owen, and Collier.” Bulletin of Marine Science 1, no. 1: 72–87.

[mec70457-bib-0075] Marshall, D. A. , N. C. Coxe , M. K. La Peyre , et al. 2021. “Tolerance of Northern Gulf of Mexico Eastern Oysters to Chronic Warming at Extreme Salinities.” Journal of Thermal Biology 100: 103072. 34503809 10.1016/j.jtherbio.2021.103072

[mec70457-bib-0076] O'Beirn, F. X. , and R. L. Walker . 1999. “Pea Crab, Pinna Theres Ostreum Say, 1817, in the Eastern Oyster, *Crassostrea virginica* (Gmelin, 1791): Prevalence and Apparent Adverse Effects on Oyster Gonad Development.” Veliger 42, no. 1: 17–20.

[mec70457-bib-0077] Oksanen, J. , G. Simpson , F. Blanchet , et al. 2025. “vegan: Community Ecology Package.” R Package Version 2.8‐0. https://vegandevs.github.io/vegan/.

[mec70457-bib-0078] Orr, H. A. 2009. “Fitness and Its Role in Evolutionary Genetics.” Nature Reviews Genetics 10, no. 8: 531–539. 10.1038/nrg2603 PMC2753274 19546856

[mec70457-bib-0079] Pebesma, E. J. 2004. “Multivariable Geostatistics in S: The gstat Package.” Computers & Geosciences 30, no. 7: 683–691.

[mec70457-bib-0080] Piesz, J. L. , A. K. Scro , R. Corbett , K. M. Lundgren , R. Smolowitz , and M. Gomez‐Chiarri . 2022. “Development of a Multiplex qPCR for the Quantification of Three Protozoan Parasites of the Eastern Oyster *Crassostrea virginica* .” Diseases of Aquatic Organisms 151: 111–121. 36300764 10.3354/dao03694

[mec70457-bib-0081] Pritchard, D. W. 1952. “Salinity Distribution and Circulation in the Chesapeake Bay Estuarine System.” Journal of Marine Research 11, no. 2: 106–123.

[mec70457-bib-0082] Privé, F. , H. Aschard , A. Ziyatdinov , and M. G. B. Blum . 2018. “Efficient Analysis of Large‐Scale Genome‐Wide Data With Two R Packages: Bigstatsr and Bigsnpr.” Bioinformatics (Oxford, England) 34, no. 16: 2781–2787. 29617937 10.1093/bioinformatics/bty185 PMC6084588

[mec70457-bib-0083] Proestou, D. A. , B. T. Vinyard , R. J. Corbett , et al. 2016. “Performance of Selectively‐Bred Lines of Eastern Oyster, *Crassostrea virginica* , Across Eastern US Estuaries.” Aquaculture (Amsterdam, Netherlands) 464: 17–27.

[mec70457-bib-0084] Puritz, J. B. , H. Zhao , X. Guo , et al. 2022. “Nucleotide and Structural Polymorphisms of the Eastern Oyster Genome Paint a Mosaic of Divergence, Selection, and Human Impacts.” *bioRxiv* 2022.08.29.505629. 10.1101/2022.08.29.505629.

[mec70457-bib-0085] Ragone Calvo, L. M. , G. W. Calvo , and E. M. Burreson . 2003. “Dual Disease Resistance in a Selectively Bred Eastern Oyster, *Crassostrea virginica* , Strain Tested in Chesapeake Bay.” Aquaculture (Amsterdam, Netherlands) 220, no. 1–4: 69–87.

[mec70457-bib-0086] Randall, C. J. , and R. van Woesik . 2015. “Contemporary White‐Band Disease in Caribbean Corals Driven by Climate Change.” Nature Climate Change 5, no. 4: 375–379.

[mec70457-bib-0087] Ray, S. M. 1952. “A Culture Technique for the Diagnosis of Infections With *Dermocystidium marinum* Mackin, Owen, and Collier in Oysters.” Science (New York, N.Y.) 116, no. 3014: 360–361. 12984123 10.1126/science.116.3014.360

[mec70457-bib-0088] Rêgo, A. , J. Baur , C. Girard‐Tercieux , M. de la Paz Celorio‐Mancera , R. Stelkens , and D. Berger . 2025. “Repeatability of Evolution and Genomic Predictions of Temperature Adaptation in Seed Beetles.” Nature Ecology & Evolution 9, no. 6: 1061–1074. 40379980 10.1038/s41559-025-02716-5 PMC12148939

[mec70457-bib-0089] Rellstab, C. , B. Dauphin , and M. Exposito‐Alonso . 2021. “Prospects and Limitations of Genomic Offset in Conservation Management.” Evolutionary Applications 14, no. 5: 1202–1212. 34025760 10.1111/eva.13205 PMC8127717

[mec70457-bib-0090] Rellstab, C. , F. Gugerli , A. J. Eckert , A. M. Hancock , and R. Holderegger . 2015. “A Practical Guide to Environmental Association Analysis in Landscape Genomics.” Molecular Ecology 24, no. 17: 4348–4370. 26184487 10.1111/mec.13322

[mec70457-bib-0091] Rellstab, C. , S. Zoller , L. Walthert , et al. 2016. “Signatures of Local Adaptation in Candidate Genes of Oaks (*Quercus* spp.) With Respect to Present and Future Climatic Conditions.” Molecular Ecology 25, no. 23: 5907–5924. 27759957 10.1111/mec.13889

[mec70457-bib-0092] Rhoné, B. , D. Defrance , C. Berthouly‐Salazar , et al. 2020. “Pearl Millet Genomic Vulnerability to Climate Change in West Africa Highlights the Need for Regional Collaboration.” Nature Communications 11, no. 1: 5274. 10.1038/s41467-020-19066-4 PMC7573578 33077747

[mec70457-bib-0093] Riginos, C. , E. D. Crandall , L. Liggins , P. Bongaerts , and E. A. Treml . 2016. “Navigating the Currents of Seascape Genomics: How Spatial Analyses Can Augment Population Genomic Studies.” Current Zoology 62, no. 6: 581–601. 29491947 10.1093/cz/zow067 PMC5804261

[mec70457-bib-0094] Rollins, J. L. 2017. “Body‐Size and Growth‐Rate Divergence Among Populations of Threespine Stickleback ( *Gasterosteus aculeatus* ) in Cook Inlet, Alaska, USA.” Canadian Journal of Zoology 95, no. 11: 877–884.

[mec70457-bib-0095] Rowley, A. F. , C. Baker‐Austin , A. S. Boerlage , et al. 2024. “Diseases of Marine Fish and Shellfish in an Age of Rapid Climate Change.” iScience 27, no. 9: 110838. 39318536 10.1016/j.isci.2024.110838 PMC11420459

[mec70457-bib-0096] Roy, B. A. , and J. W. Kirchner . 2000. “Evolutionary Dynamics of Pathogen Resistance and Tolerance.” Evolution 54, no. 1: 51–63. 10937183 10.1111/j.0014-3820.2000.tb00007.x

[mec70457-bib-0097] Rundle, H. D. , S. M. Vamosi , and D. Schluter . 2003. “Experimental Test of Predation's Effect on Divergent Selection During Character Displacement in Sticklebacks.” Proceedings of the National Academy of Sciences of the United States of America 100, no. 25: 14943–14948. 14630946 10.1073/pnas.2036360100 PMC299857

[mec70457-bib-0098] Sanford, E. , and M. W. Kelly . 2011. “Local Adaptation in Marine Invertebrates.” Annual Review of Marine Science 3, no. 1: 509–535. 10.1146/annurev-marine-120709-142756 21329215

[mec70457-bib-0099] Schaeffer, M. S. , and E. E. Levitt . 1956. “Concerning Kendall's Tau, a Nonparametric Correlation Coefficient.” Psychological Bulletin 53, no. 4: 338–346. 13336201 10.1037/h0045013

[mec70457-bib-0101] Smith, T. B. , M. T. Kinnison , S. Y. Strauss , T. L. Fuller , and S. P. Carroll . 2014. “Prescriptive Evolution to Conserve and Manage Biodiversity.” Annual Review of Ecology, Evolution, and Systematics 45, no. 1: 1–22.

[mec70457-bib-0102] Urban, M. C. 2015. “Climate Change. Accelerating Extinction Risk From Climate Change.” Science (New York, N.Y.) 348, no. 6234: 571–573. 25931559 10.1126/science.aaa4984

[mec70457-bib-0103] Varas‐Myrik, A. , F. Sepúlveda‐Espinoza , Ó. Toro‐Núñez , A. Fajardo , D. Alarcón , and R. Hasbún . 2024. “Using a Genomic Offset Approach to Guide Assisted Gene Flow in the South American Conifer *Araucaria araucana* .” Forest Ecology and Management 553: 121637.

[mec70457-bib-0104] Verrico, B. M. , T. Capblancq , M. C. Fitzpatrick , and S. R. Keller . 2025. “Reciprocal Evaluation of Genomic Offset Predictions of Climate Maladaptation With Independent Empirical Datasets.” American Naturalist 207, no. 3: 415–432. 10.1086/739111 41730224

[mec70457-bib-0105] Wadgymar, S. M. , M. L. DeMarche , E. B. Josephs , S. N. Sheth , and J. T. Anderson . 2022. “Local Adaptation: Causal Agents of Selection and Adaptive Trait Divergence.” Annual Review of Ecology, Evolution, and Systematics 53, no. 1: 87–111. 10.1146/annurev-ecolsys-012722-035231 PMC10544833 37790997

[mec70457-bib-0106] Wadgymar, S. M. , S. Sheth , E. Josephs , M. DeMarche , and J. Anderson . 2024. “Defining Fitness in Evolutionary Ecology.” International Journal of Plant Sciences 185, no. 3: 218–227. 39035046 10.1086/729360 PMC11257499

[mec70457-bib-0107] Waldvogel, A.‐M. , B. Feldmeyer , G. Rolshausen , et al. 2020. “Evolutionary Genomics Can Improve Prediction of Species' Responses to Climate Change.” Evolution Letters 4, no. 1: 4–18. 32055407 10.1002/evl3.154 PMC7006467

[mec70457-bib-0108] Weir, B. S. , and C. C. Cockerham . 1984. “Estimating F‐Statistics for the Analysis of Population Structure.” Evolution 38, no. 6: 1358–1370. 28563791 10.1111/j.1558-5646.1984.tb05657.x

[mec70457-bib-0109] Wells, H. W. 1961. “The Fauna of Oyster Beds, With Special Reference to the Salinity Factor.” Ecological Monographs 31, no. 3: 239–266.

[mec70457-bib-0110] Zu Ermgassen, P. S. E. , M. D. Spalding , B. Blake , et al. 2012. “Historical Ecology With Real Numbers: Past and Present Extent and Biomass of an Imperilled Estuarine Habitat.” Proceedings of the Royal Society B: Biological Sciences 279, no. 1742: 3393–3400. 10.1098/rspb.2012.0313 PMC3396889 22696522

