# Supplementary material for: Genomic Offsets Predict Survival With Low Accuracy in a Marine Common Garden
Source: Mol Ecol. 2026 Jul 4;35(13):e70457. doi: 10.1111/mec.70457 (PMC13332419; doi:10.1111/mec.70457)
Supplement: Supplementary file 1 — File S1: Experimental oyster survival. File S2: Experimental oyster lengths. File S3: Offsets from experimental populations to common gardens. File S4: Offsets from seascape populations to common gardens. Figure S1: Disease pressure during the experimental period at both common garden sites. (A) MSX prevalence through time. (B) Dermo prevalence through time. (C) Median prevalence of MSX, Dermo and pea crabs during the common garden experiment. Figure S2: (A) Salinity, measured in parts per thousand, and (B) temperature, measured in degrees Celsius, at common garden sites over the experimental period. Figure S3: Environmental distances between source population sites‐of‐origin and common gardens, calculated as Euclidean distance. Figure S4: Pairwise F ST of wild experimental source populations. Figure S5: Fitness‐Proxies throughout the common garden experiment. Each data point represents population level survival or length averages at each monitoring event. Figure S6: Correlations between predicted offset and experimental survival thru time. LEAoffset, RDAoffset, GFoffset and environmental distance are all shown. Figure S7: Correlations between predicted offset and experimental length through time. LEAoffset, RDAoffset, GFoffset and environmental distance are all shown. Figure S8: Correlations between offset predicted by different methods. Comparisons are between LEAoffset, RDAoffset, GFoffset and environmental distance at each common garden site. Correlations reported are Kendall's Tau. Table S1: Geographic information about populations included in the seascape training dataset. Table S2: Population genetic statistics of the six wild experimental source populations, including expected (H e) and observed (H o) heterozygosity, as well as inbreeding coefficient (F IS). Table S3: Experimental fitness correlation with genomic offset, with and without disease, calculated without inclusion of selection lines. [file MEC-35-e70457-s001.zip › mec70457-sup-0005-FigureS1-S8-TableS1-S3@Supplement.docx]

# Supplement for “*Genomic Offsets Predict Survival with Low Accuracy in a Marine Common Garden*”

[**Supplement for “Genomic Offsets Predict Survival with Low Accuracy in a Marine Common Garden” 1**](#_8ucp9novojp8)

[Supplemental methods 2](#_7m4diuyakkhj)

[qPCR for Disease Quantification 2](#_svgkxlyv4cbk)

[Supplemental Files 2](#_uy443hwncy4w)

[​​Supp. file 1: Experimental Oyster Survival 3](#_uwuhp8imtwbj)

[​​Supp. file 2: Experimental Oyster Lengths 3](#_vt7uq1gcavey)

[​​Supp. file 3: Offsets from Experimental Populations to Common Gardens 3](#_k9p3dyzfjpx3)

[​​Supp. file 4: Offsets from Seascape Populations to Common Gardens 3](#_gbdgsrtt3kyw)

[Supplemental Tables 4](#_yjcvovecvwc5)

[Supp. Table S1. Populations included in the Seascape Training Dataset](#_lj3opby9dl9n) 5

Supp. Table S2. Population Genetic Statistics of Wild Experimental Source Populations 6

Supp. Table S3. Experimental Fitness Correlation with Genomic Offset, with and without Disease, No Selection Lines 7

[Supplemental Figures](#_47i5z5p4n4q4) 8

[Fig. S1. Disease during Experimental Period](#_ebf5nvgvxpay) 8

[Fig. S2. Abiotic Conditions at Common Garden Sites over Experimental Period](#_tbpu7d196hcw) 9

[Fig. S3. Environmental Distances between Source Population Sites-of-Origin & Common](#_14x69qdyztv)  10

[Gardens](#_14x69qdyztv)

Fig. S4. Pairwise F_ST_ of Experimental Source Populations 11

[Fig. S5. Fitness-Proxies throughout the Common Garden Experiment](#_xay56gq0l7j3) 12

[Fig. S6. Correlations between Predicted Offset and Experimental Survival thru Time](#_6up7volc6qwt) 13

[Fig. S7. Correlations between Predicted Offset and Experimental Length thru Time](#_t502lu7dgul7) 15

[Fig. S8. Correlations between Offset Predicted by Different Methods](#_m17fk56vwqq5) 17

##

## Supplemental methods

### *qPCR for Disease Quantification*

Eastern oyster tissue samples (n=20 per site) were collected during a single sampling day per sampling site during a peak disease month (June - October) in 2022. Host genomic and parasite DNA were co-extracted from 18-22 mg of gill tissue using the Qiagen DNeasy Blood and Tissue kit protocol eluted in low TE (can reduce info if needed and cite seascape).

Extracted DNA was amplified to assess prevalence of the parasites Perkinsus marinus (causative agent of Dermo) and Haplosporidium nelsoni (causative agent of MSX) using a duplex quantitative polymerase chain reaction (qPCR) following the methods of Piesz et al (2022). We used MSX primers from Wilbur et al (2012) and Dermo primers from De Faveri et al (2009). Each 10 μl reaction consisted of 1 μl template DNA, 5.56 μl TaqMan Multiplex MasterMix, 3.33 μl molecular grade H20, 0.56 μl 20x Dermo primer/probe mix, and 0.56 μl 20x MSX primer/probe mix. The 20x primer/probe mix for each parasite consisted of 18 μl each of forward and reverse primers, 59 μl of 1x TE buffer, and 5 μl of TaqMan MGB probe for MSX, 5 μl of TaqMan QSY probe for Dermo, respectively.

Standard curves were generated using synthetic gBlock gene fragments (Integrated DNA Technologies) containing target regions for each parasite. gBlocks were resuspended in 1x TE buffer and diluted to 1 ng/μl. Equal volumes of MSX and Dermo gBlock solutions were serially diluted 1:10 in 1x TE buffer to generate a standard series ranging from 0.1 ng/μl to 10^-7^ ng/μl. For each plate, standard curves were constructed by retaining a minimum of five concentrations, excluding the highest concentration which is not biologically representative of field samples. Positive controls were obtained from concentrated and dried DNA isolated by the Shellfish Pathology Laboratory at the Virginia Institute of Marine Science, rehydrated in low TE. qPCR cycling conditions included initial denaturation at 95℃ for 30 sec, followed by 40 cycles of 95℃ for 10 sec and 60℃ for 30 sec. Samples were analyzed on a Bio-Rad CFX96 Real-Time System with Bio-Rad CFX Maestro software.

Standard curves were required to pass a threshold of R^2^ ≥ 0.990 to retain the plate for analysis. All standards, samples, positive controls, and negative controls were run in duplicate; if any sample differed in amplification by >2 Cq (Quantification Cycle), or were inconsistent (one amplified and one did not) they were re-run to confirm presence or absence. Further, to limit the likelihood of false positives or detection of primer-dimer formation at low template concentrations, the criteria for a sample to be considered infected (positive) also required a Cq value <27 for detection of MSX or a Cq value <37 for Dermo. These Cq thresholds were more conservative than those used in similar studies (e.g., Hanley et al 2023 where Cq cutoff was <38 for both parasites; Piesz et al 2022 where Cq cutoff was <31 for MSX and <36 for Dermo; Carrasquillo et al. 2024 where Cq cutoff was <39 for both MSX and Dermo). Cq thresholds were chosen to avoid likely false detections of MSX in Gulf coast populations, where MSX has never been documented (Burreson 2008, Ford et al. 2011). Population-level disease prevalence was calculated as the percentage of oysters that were positive for each parasite.

**Supplemental References**:

Burreson, E. M. (2008). Misuse of PCR assay for diagnosis of mollusc protistan infections. *Diseases of Aquatic Organisms*, *80*(1), 81–83.

Carrasquillo, A., Proestou, D. A., Hudson, R., Preziosi, B., & Gomez-Chiarri, M. (2024). Genotypic and phenotypic analysis of eastern oyster populations for use in aquaculture. Journal of Shellfish Research, 43(3), 421–442.

Faveri, J. D., Smolowitz, R. M., & Roberts, S. B. (2009). Development and validation of a real-time quantitative PCR assay for the detection and quantification ofPerkinsus marinus in the Eastern oyster,Crassostrea virginica. Journal of Shellfish Research, 28(3), 459–464.

Ford, S. E., Paterno, J., Scarpa, E., Stokes, N. A., Kim, Y., Powell, E. N., & Bushek, D. (2011). Widespread survey finds no evidence of Haplosporidium nelsoni (MSX) in Gulf of Mexico oysters. *Diseases of Aquatic Organisms*, *93*(3), 251–256.

Hanley, T. C., Grabowski, J. H., Schneider, E. G., Barrett, P. D., Puishys, L. M., Spadafore, R., McManus, G., Helt, W. S. K., Kinney, H., Conor McManus, M., & Randall Hughes, A. (2023). Host genetic identity determines parasite community structure across time and space in oyster restoration. Proceedings. Biological Sciences, 290(1995), 20222560.

Piesz, J. L., Scro, A. K., Corbett, R., Lundgren, K. M., Smolowitz, R., & Gomez-Chiarri, M. (2022). Development of a multiplex qPCR for the quantification of three protozoan parasites of the eastern oyster Crassostrea virginica. Diseases of Aquatic Organisms, 151, 111–121.

Wilbur, A. E., Ford, S. E., Gauthier, J. D., & Gomez-Chiarri, M. (2012). Quantitative PCR assay to determine prevalence and intensity of MSX (Haplosporidium nelsoni) in North Carolina and Rhode Island oysters Crassostrea virginica. Diseases of Aquatic Organisms, 102(2), 107–118.

## Supplemental Files

### ​​Supp. file 1: Experimental Oyster Survival

### ​​Supp. file 2: Experimental Oyster Lengths

### ​​Supp. file 3: Offsets from Experimental Populations to Common Gardens

### ​​Supp. file 4: Offsets from Seascape Populations to Common Gardens

##

##

## Supplemental Tables

### *Supp. Table S1. Populations included in the Seascape Training Dataset*

| **Table S1: Populations included in the Seascape Training Dataset** | | | | | |
| --- | --- | --- | --- | --- | --- |
| ***Relative***  ***Geographic***  ***Region*** | ***Ancestral***  ***Group*** | ***Location*** | ***Site Abbreviation*** | ***Environment-of-***  ***origin Latitude*** | ***Environment-of-***  ***origin Longitude*** |
| Gulf | Gulf | Copano Bay Reef, TX | TX1 | 28.096 | -97.174 |
| Gulf | Gulf | Tres Palacios, TX | TX2 | 28.6936 | -96.2233 |
| Gulf | Gulf | Galveston Bay, TX | TX3 | 29.61167 | -94.9586 |
| Gulf | Gulf | Sabine Lake, LA | LA1 | 29.785 | -93.9181 |
| Gulf | Gulf | 09 Cultch, LA | LA2 | 29.84499 | -93.3179 |
| Gulf | Gulf | Nickle Reef, LA | LA3 | 29.4196 | -91.7072 |
| Gulf | Gulf | Sister Lake, LA | LA4 | 29.23992 | -90.9114 |
| Gulf | Gulf | 2008 Culch Plant, LA | LA5 | 29.42362 | -90.0191 |
| Gulf | Gulf | Lake Fortuna, LA | LA6 | 29.64379 | -89.4976 |
| Gulf | Gulf | Cedar Point Reef, AL | AL1 | 30.37278 | -88.3183 |
| Gulf | Gulf | St Vincent Sound, FL | FL1 | 29.68911 | -85.2207 |
| Gulf | Gulf | East Cove, FL | FL2 | 29.69568 | -84.7893 |
| Southeast | Atlantic | Timucuan Preserve, FL | FL3 | 30.44003 | -81.4364 |
| Southeast | Atlantic | Sapelo Island, GA | GA1 | 31.41777 | -81.2958 |
| Southeast | Atlantic | Meridian Ferry Dock, GA | GA2 | 31.45351 | -81.3632 |
| Southeast | Atlantic | Skidaway, GA | GA3 | 31.98995 | -81.0217 |
| Southeast | Atlantic | Fort Pulaski Oyster Creek, GA | GA4 | 32.01721 | -80.922 |
| Southeast | Atlantic | Grice Cove, SC | SC1 | 32.75231 | -79.8977 |
| Southeast | Atlantic | Oyster Landing, SC | SC2 | 33.52353 | -79.0618 |
| Southeast | Atlantic | Fort Fisher Jetty, NC | NC1 | 33.95869 | -77.9418 |
| Southeast | Atlantic | UNCW Dock, NC | NC2 | 34.14023 | -77.8636 |
| Mid-Atlantic | Atlantic | Custis Channel, VA | VA1 | 37.61662 | -75.6639 |
| Mid-Atlantic | Atlantic | Lower Cedar Point, MD1 | MD1 | 38.33696 | -76.9767 |
| Mid-Atlantic | Atlantic | Cape Shore, NJ1 | NJ1 | 39.07359 | -74.913 |
| Mid-Atlantic | Atlantic | Hope Creek, NJ2 | NJ2 | 39.43472 | -75.5169 |
| Northeast | Atlantic | Ash Creek, CT | CT1 | 41.14643 | -73.2358 |
| Northeast | Atlantic | Duck Cove, RI | RI1 | 41.55897 | -71.4379 |
| Northeast | Atlantic | Sawyer’s Island, MA | MA1 | 42.75173 | -70.8366 |
| Northeast | Atlantic | Mouth of Squamscott River, NH | NH1 | 43.05375 | -70.9115 |
| Northeast | Atlantic | Orrs Cove, ME | ME1 | 43.83136 | -69.9153 |
| Northeast | Atlantic | Days Cove, ME | ME2 | 44.02452 | -69.532 |
| Northeast | Atlantic | Salt Bay, Canada | CN1 | 43.81249 | -65.9106 |

##

### *Table S2. Population Genetic Statistics of Wild Experimental Source Populations*

| **Table S2: Population Genetic Statistics of Wild Experimental Source Populations** | | | | | |
| --- | --- | --- | --- | --- | --- |
| ***Population*** | ***Ancestral Group*** | ***Relative Geographic Region*** | ***H_O_*** | ***H_E_*** | ***F_IS_*** |
| W1-TX | Gulf | Gulf | 0.211 | 0.254 | 0.154 |
| W2-LA | Gulf | Gulf | 0.212 | 0.255 | 0.155 |
| W3-FL | Atlantic | Southeast | 0.223 | 0.267 | 0.158 |
| W4-VA | Atlantic | Mid-Atlantic | 0.218 | 0.256 | 0.136 |
| W5-NH | Atlantic | Northeast | 0.221 | 0.260 | 0.135 |
| W6-ME | Atlantic | Northeast | 0.216 | 0.252 | 0.132 |

### Table. S2: Population genetic statistics of wild experimental source populations, including observed heterozygosity (H_O_), expected heterozygosity (H_E_), and inbreeding coefficient (F_IS_).

### *Supp. Table S3. Experimental Fitness Correlation with Genomic Offset, with and without Disease, without Selection Lines*

| **Table S3: Experimental Fitness Correlation with Genomic Offset, with and without Disease** | | | | | | | |
| --- | --- | --- | --- | --- | --- | --- | --- |
| ***Method*** | ***Common Garden*** | ***τ_survival, no disease_*** | ***τ_survival, disease_*** | ***∆τ_survival_***  ***Disease - No Disease Model***  ***(+ is improvement)*** | ***τ_length, no disease_*** | ***τ_length, disease_*** | ***∆τ_length_***  ***Disease - No Disease Model***  ***(+ is improvement)*** |
| EnvDist | Lewisetta | -0.035 | -0.160 | +0.125 | -0.285 | -0.411 | +0.126 |
| LEA | Lewisetta | -0.160 | -0.536 | +0.376 | -0.411 | -0.591 ** | +0.180 |
| RDA | Lewisetta | +0.077 | -0.411 | +0.488 | -0.035 | -0.522 ** | +0.487 |
| GF | Lewisetta | -0.035 | -0.035 | 0.000 | -0.285 | -0.285 | 0.000 |
| EnvDist | York River | -0.018 | -0.018 | 0.000 | +0.124 | +0.124 | 0.000 |
| LEA | York River | -0.213 | -0.106 | -0.107 | -0.142 | -0.178 | +0.036 |
| RDA | York River | -0.213 | -0.426 | +0.213 | +0.071 | -0.142 | +0.213 |
| GF | York River | -0.053 | -0.319 | +0.266 | -0.231 | -0.248 | +0.017 |

##

## **Table. S3**: Correlations between genomic offset and survival (left) and length (right) at Lewisetta (top) and York River (bottom), with and without disease in the model. The improvement in performance of each method, given by the difference between the fitness proxy correlation with models built with disease + abiotic variables and models built only with abiotic variables, is shown as ∆τ. Positive change indicates that disease improved method performance, shown in green, while negative change indicates that disease worsened method performance, shown in red. Note that correlations were calculated without including the selection lines S1-LOLA and S2-DEBY.

## Supplemental Figures

### *Fig. S1. Disease during Experimental Period*

##
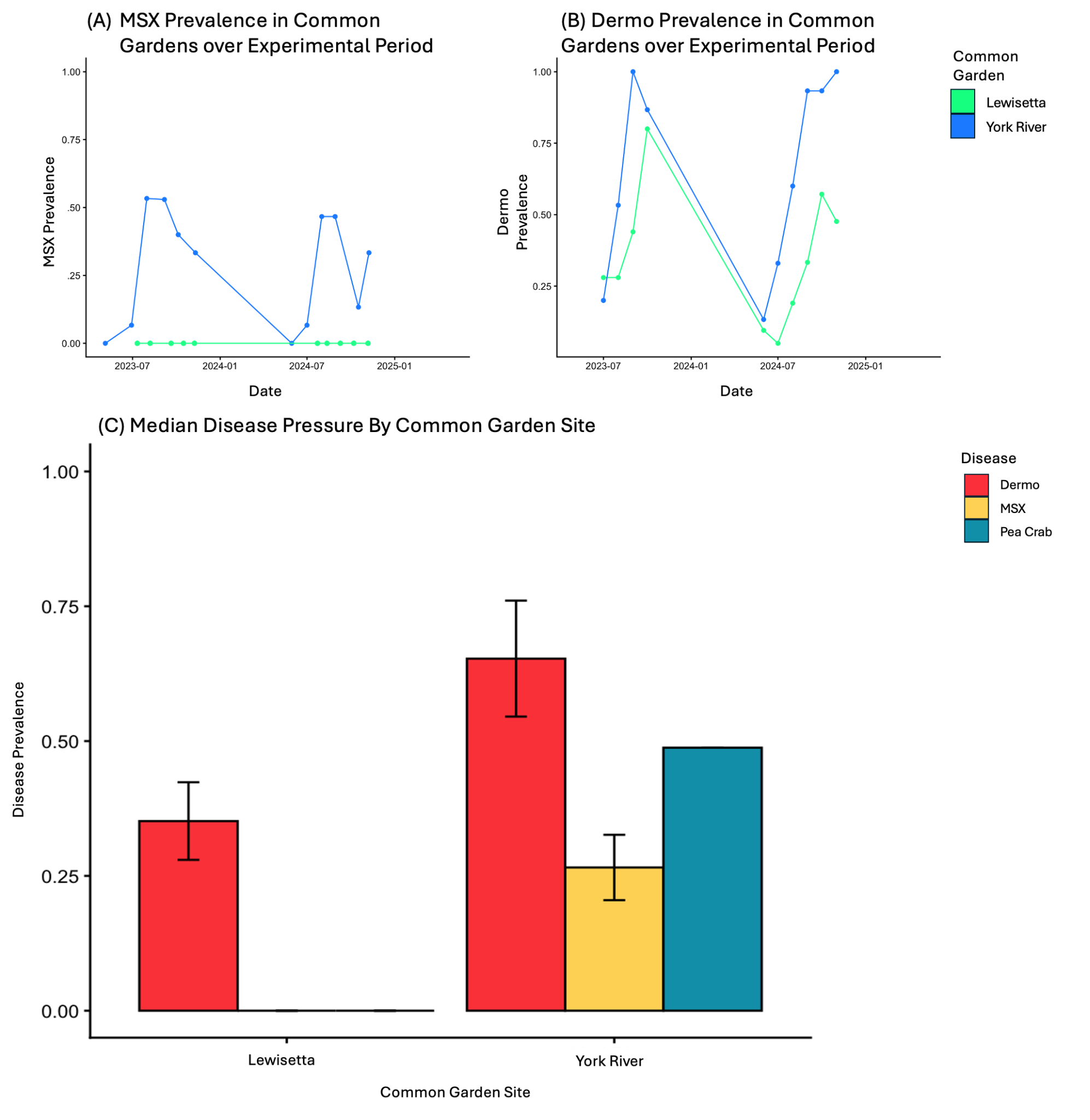


**Figure S1**. Disease at the two common garden sites during the experimental period. (A) MSX prevalence in the common gardens, and (B) Dermo prevalence in the common gardens, sampled June through October in 2023 and 2024. Lewisetta is shown in green, while York River is shown in blue. (C) Median disease pressure from Dermo (red), MSX (yellow), and pea crab (blue) infection across the experimental period. Note that pea crab prevalences were determined by a single count at the conclusion of the experiment.

### *Fig. S2. Abiotic Conditions at Common Garden Sites over Experimental Period*

##
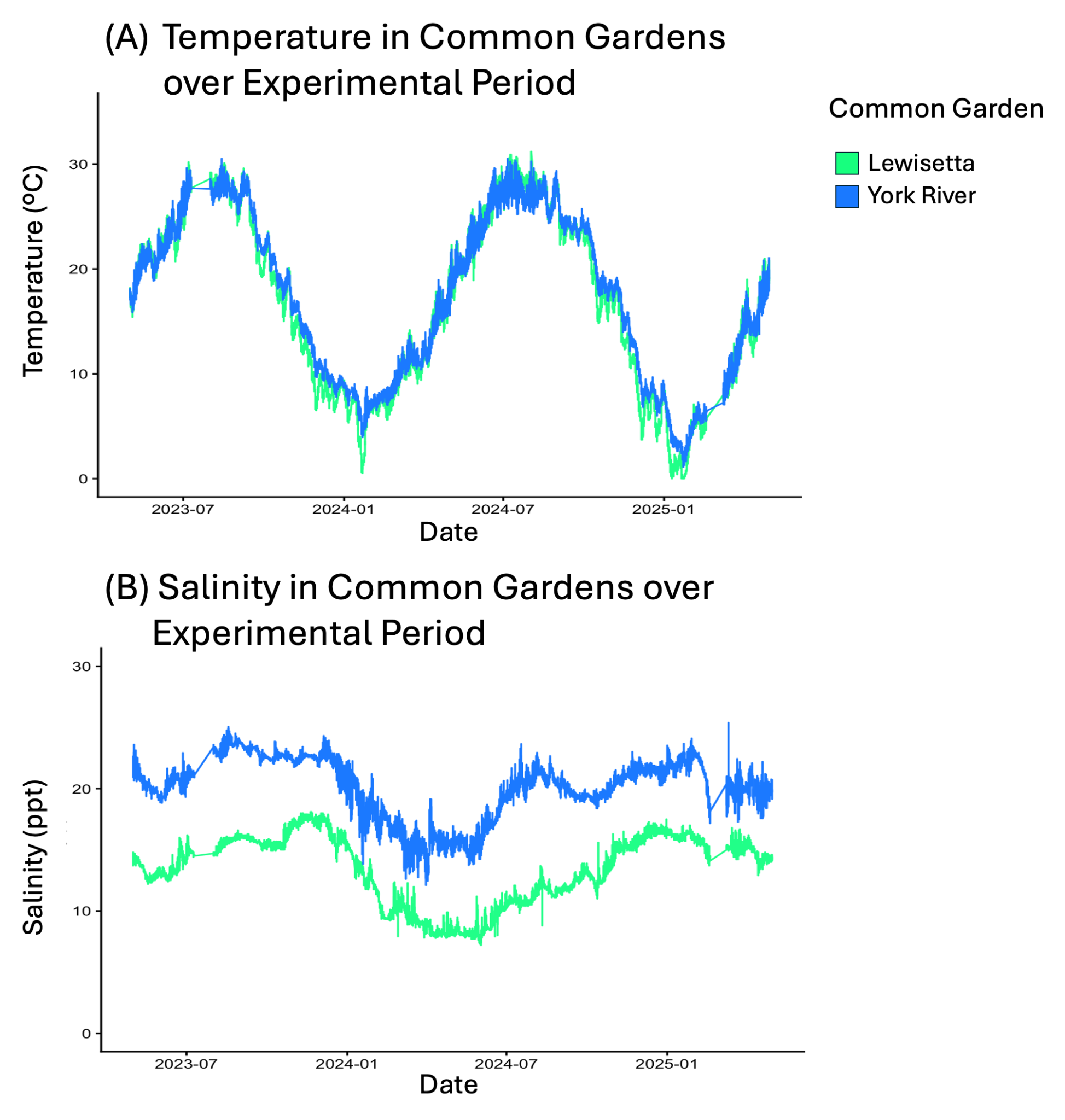


**Figure S2**. (A) Temperature (ºC) and (B) salinity (ppt) at the two common garden sites during the experimental period. Trends at Lewisetta are shown in green and trends at York River are shown in blue.

### *Fig. S3. Environmental Distances between Source Population Sites-of-Origin & Common Gardens*


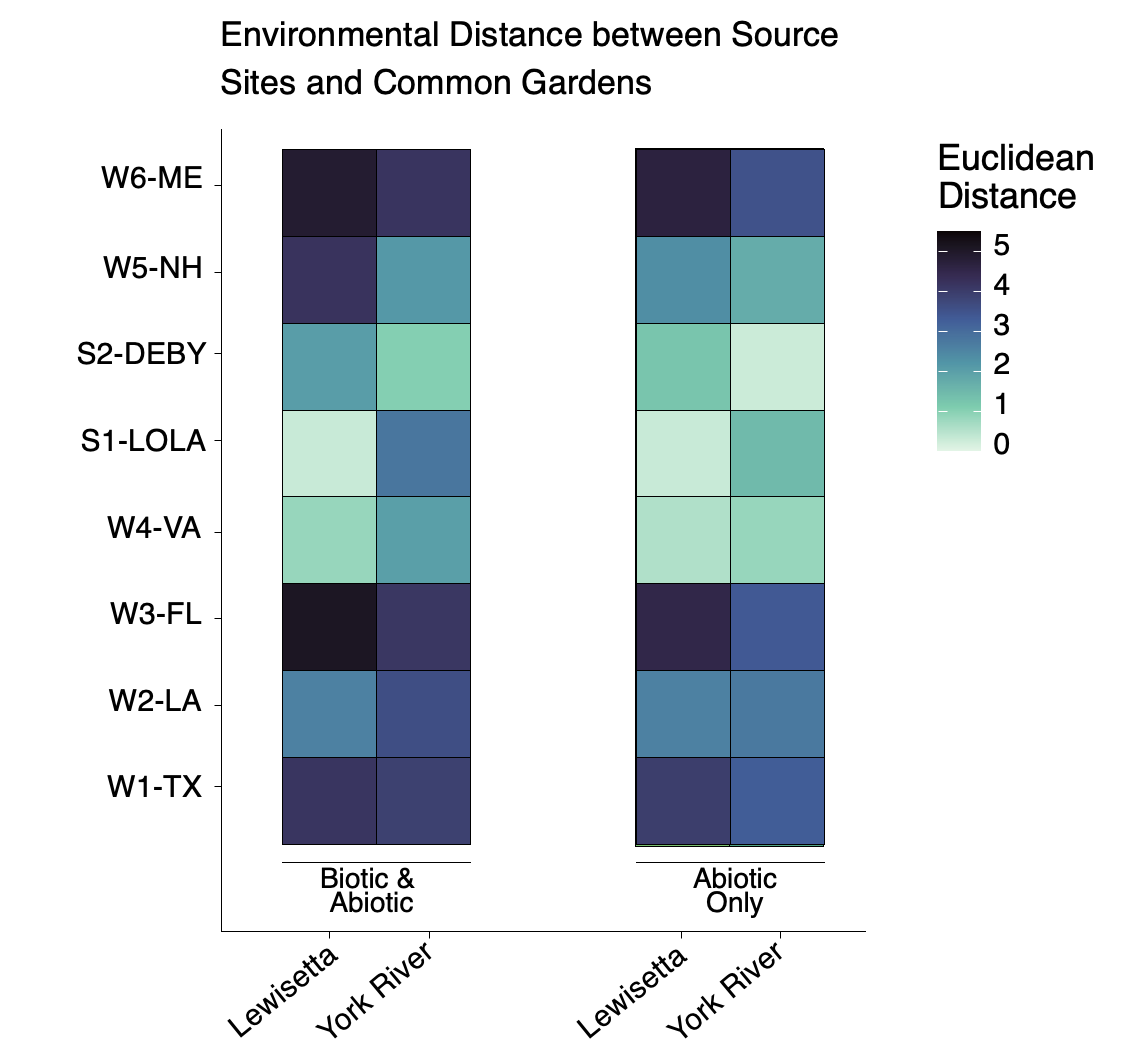


## **Figure S3**. Euclidean environmental distance between common gardens and experimental source sites with (left) and without (right) disease, specifically MSX and Dermo prevalence.

## ***Fig. S4. Pairwise F_ST_ of Experimental Source Populations***

## ***
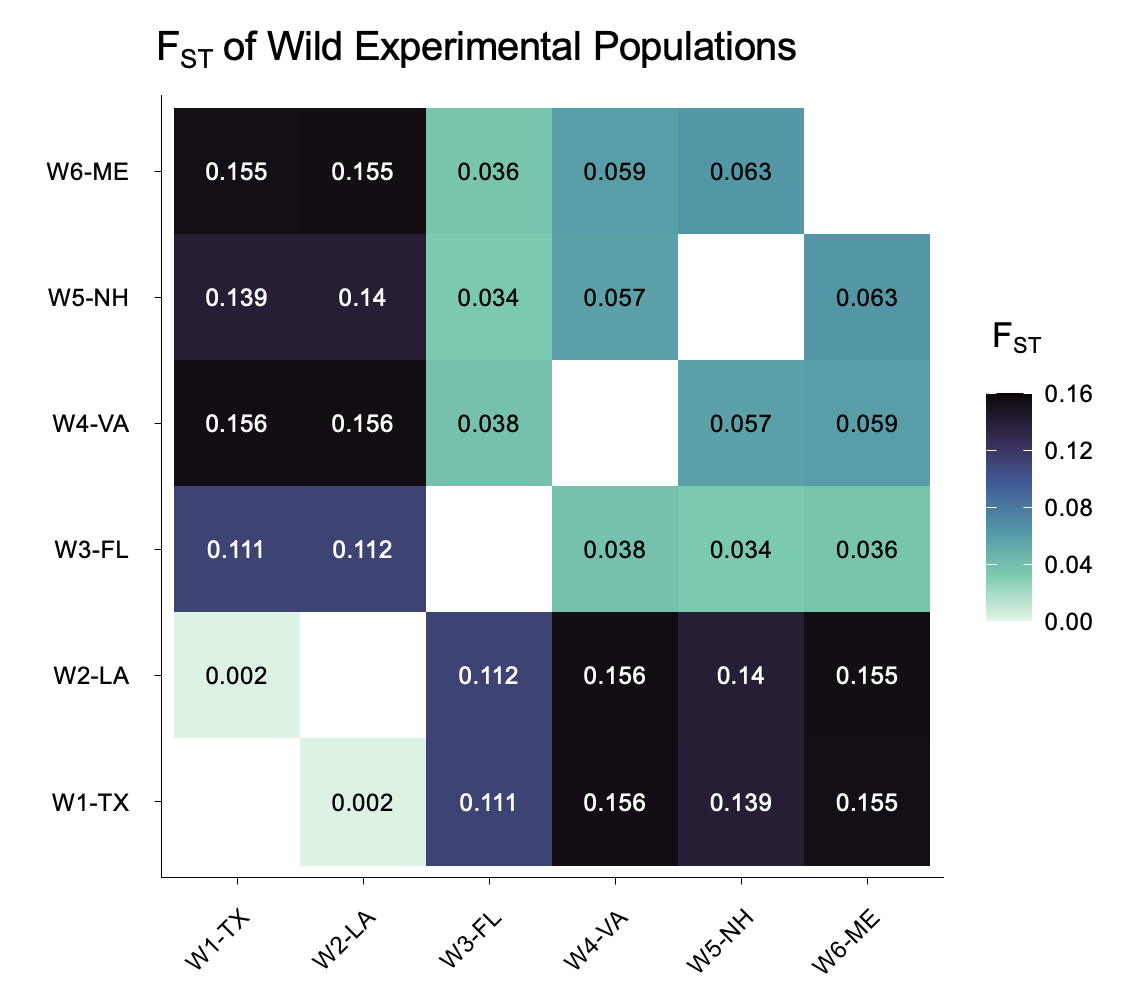
*Figure S4**. Pairwise comparisons between source populations used in experimental common gardens. Note that only wild populations, no selection lines, are shown, as FST estimates are based on seascape training genetic data.

## ***Fig. S5. Fitness-Proxies throughout the Common Garden Experiment***


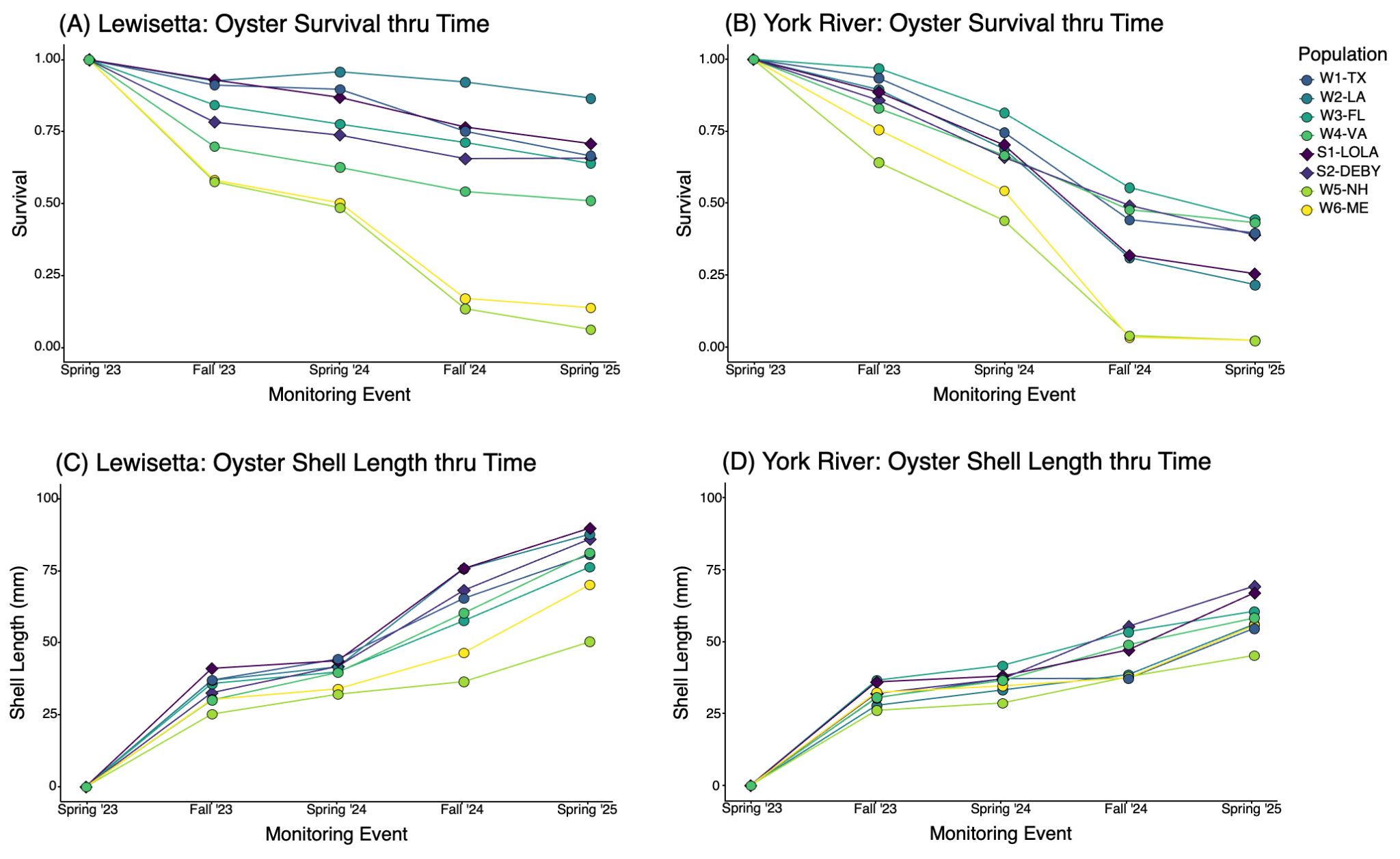


**Figure S5**. Experimental fitness-proxies through time at Lewisetta (left) and York River (right). Mean population (A-B) survival and (C-D) length at each monitoring event is shown for each experimental population.

##

## ***Fig. S6. Correlations between Predicted Offset and Experimental Survival thru Time***


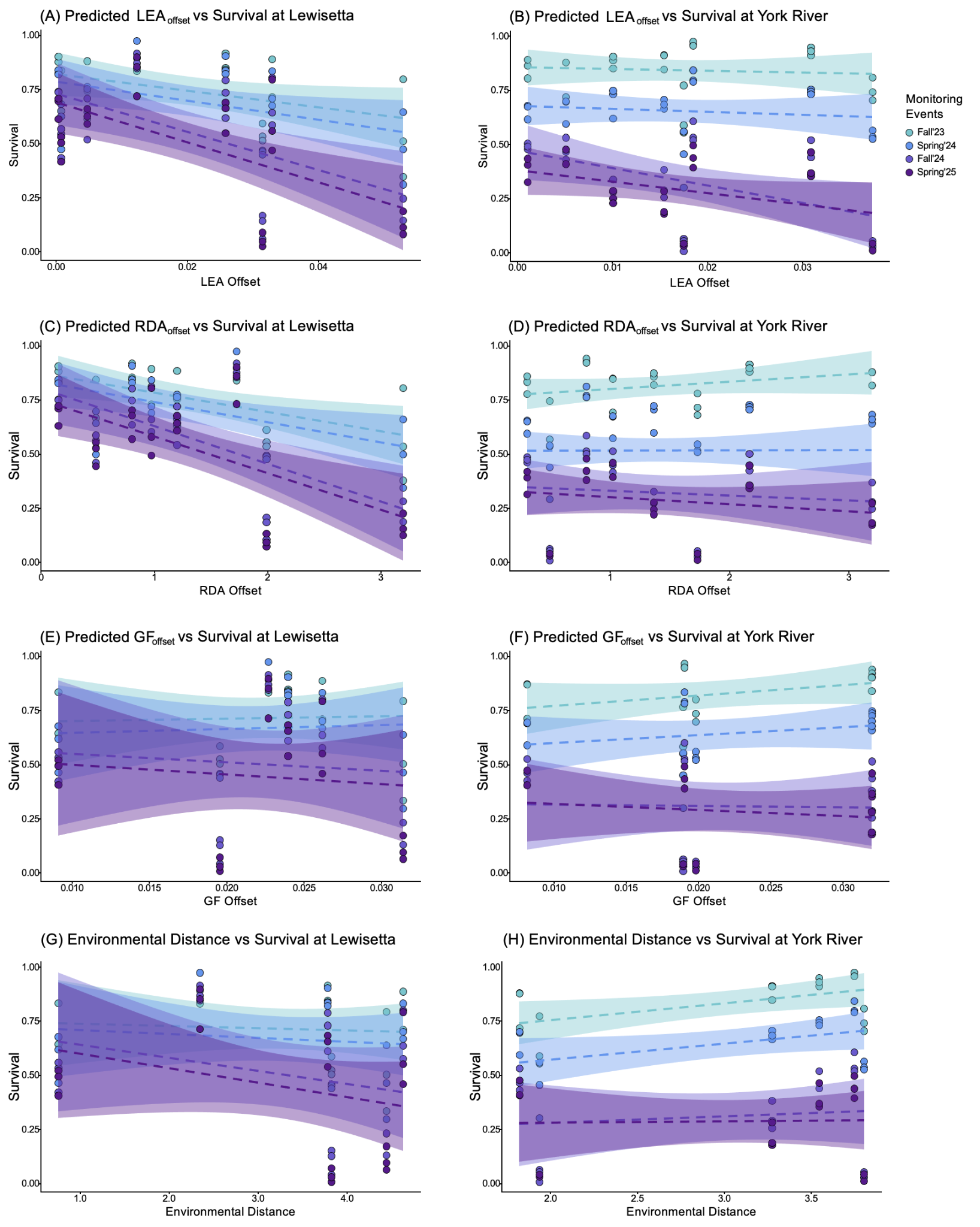


**Figure S6**. Correlations between experimental survival and (A-B) LEA_offset_ , (C-D) RDA_offset_ , (E-F) GF_offset_ and (G-H) Euclidean environmental distance. Correlations at Lewisetta are shown on the left, while those at York River are on the right. Significant correlations are indicated by solid lines, while non-significant correlations are dotted. Colors represent the monitoring event from which survival data was taken.

## ***Fig. S7. Correlations between Predicted Offset and Experimental Length thru Time***
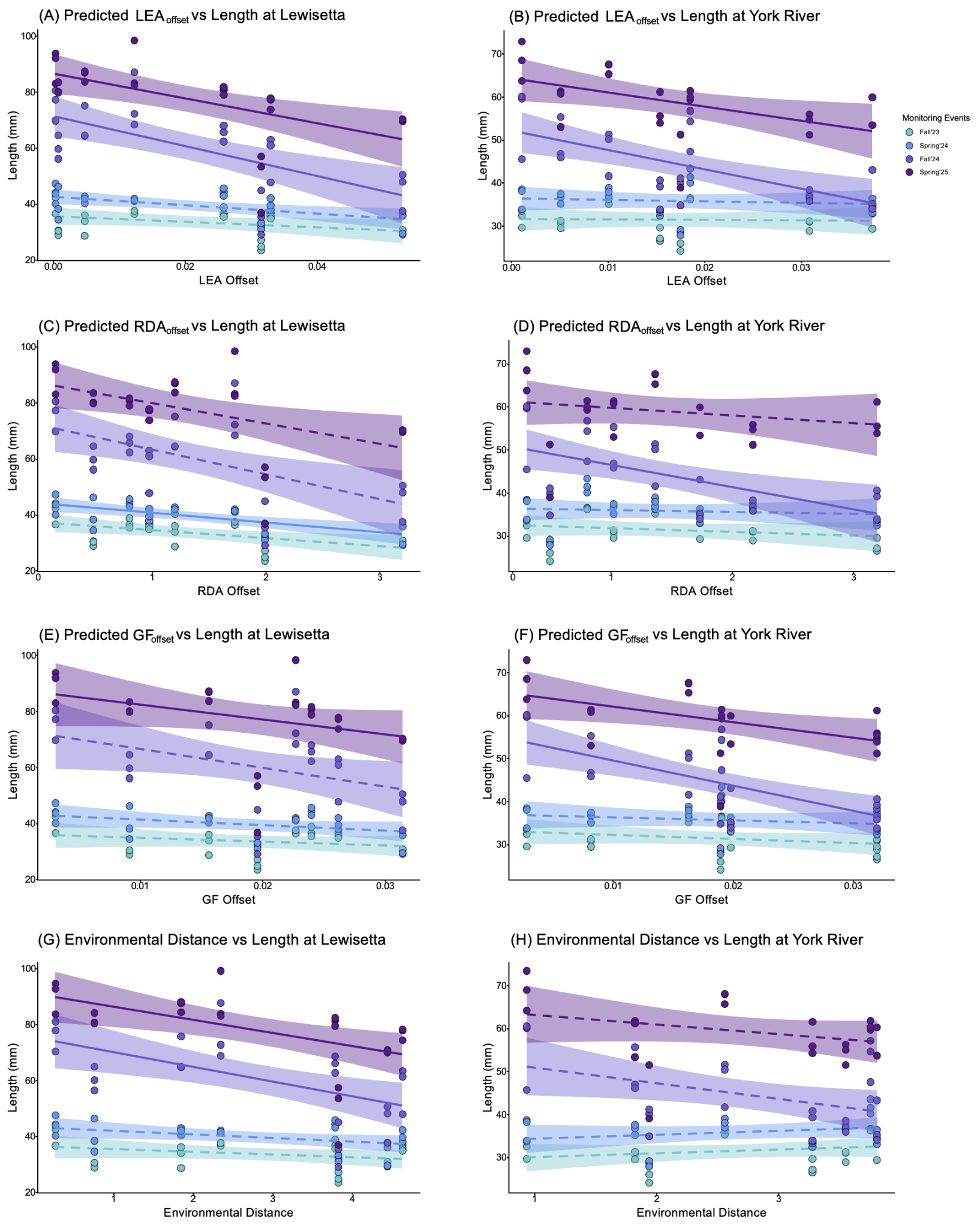


**Figure S7**. Correlations between experimental length and (A-B) LEA_offset_ , (C-D) RDA_offset_ , (E-F) GF_offset_ and (G-H) Euclidean environmental distance. Correlations at Lewisetta are shown on the left, while those at York River are on the right. Significant correlations are indicated by solid lines, while non-significant correlations are dotted. Colors represent the monitoring event from which survival data was taken.

##

## ***Fig. S8. Correlations between Offset Predicted by Different Methods***


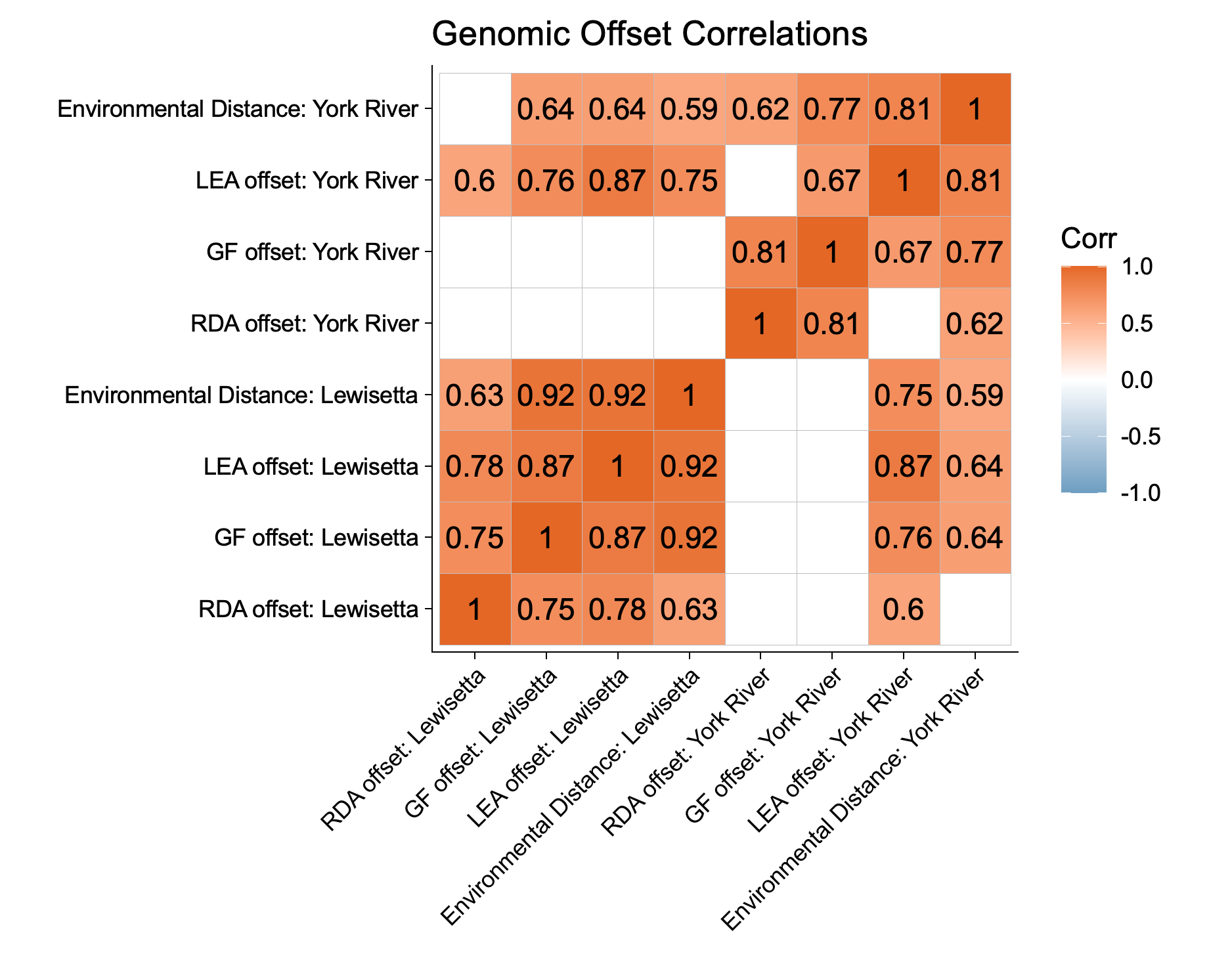


**Figure S8**. Correlations between genomic offset predictions/environmental distance made with different methods. Correlations for Lewisetta predictions are in the bottom left quadrant and those for York River are in the top right quadrant. Only significant correlations are shown.
